# Dynamic symbioses reveal pathways to coral survival through prolonged heatwaves

**DOI:** 10.1038/s41467-020-19169-y

**Published:** 2020-12-08

**Authors:** Danielle C. Claar, Samuel Starko, Kristina L. Tietjen, Hannah E. Epstein, Ross Cunning, Kim M. Cobb, Andrew C. Baker, Ruth D. Gates, Julia K. Baum

**Affiliations:** 1 Department of Biology, University of Victoria, PO Box 1700 Station CSC, Victoria, BC V8W 2Y2 Canada; 2 School of Aquatic and Fishery Sciences, University of Washington, 1122 NE Boat St, Seattle, WA 98105 USA; 3 Department of Microbiology, Oregon State University, Corvallis, OR 97331 USA; 4 Department of Marine Biology and Ecology, Rosenstiel School of Marine and Atmospheric Science, University of Miami, 4600 Rickenbacker Causeway, Miami, FL 33149 USA; 5 Daniel P. Haerther Center for Conservation and Research, John G. Shedd Aquarium, 1200 South Lake Shore Drive, Chicago, IL 60605 USA; 6 School of Earth and Atmospheric Sciences, Georgia Institute of Technology, Atlanta, GA 30306 USA; 7 Hawaiʻi Institute of Marine Biology, 46-007b Lilipuna Road, Kāneʻohe, HI 96744 USA

**Keywords:** Climate-change ecology, Community ecology, Marine biology

## Abstract

Prospects for coral persistence through increasingly frequent and extended heatwaves seem bleak. Coral recovery from bleaching is only known to occur after temperatures return to normal, and mitigation of local stressors does not appear to augment coral survival. Capitalizing on a natural experiment in the equatorial Pacific, we track individual coral colonies at sites spanning a gradient of local anthropogenic disturbance through a tropical heatwave of unprecedented duration. Unexpectedly, some corals survived the event by recovering from bleaching while still at elevated temperatures. These corals initially had heat-sensitive algal symbiont communities, endured bleaching, and then recovered through proliferation of heat-tolerant symbionts. This pathway to survival only occurred in the absence of strong local stressors. In contrast, corals in highly disturbed areas were already dominated by heat-tolerant symbionts, and despite initially resisting bleaching, these corals had no survival advantage in one species and 3.3 times lower survival in the other. These unanticipated connections between disturbance, coral symbioses and heat stress resilience reveal multiple pathways to coral survival through future prolonged heatwaves.

## Abstract (web summary)

Climate change and local anthropogenic stressors threaten the persistence of coral reefs. Here the authors track coral bleaching over the course of a heatwave and find that some colonies recovered from bleaching while high temperatures persisted, but only at sites lacking in other strong anthropogenic stressors.

## Introduction

Climate change-amplified marine heatwaves pose an imminent threat to the world’s coral reefs^1–5^. Heat stress disrupts the vital association between corals and their photosynthetic algal symbionts (family Symbiodiniaceae^6^), causing corals to bleach^7^. While small doses of thermal stress may protect corals from future heat stress by inducing thermal tolerance^4^, extreme or long-lasting thermal anomalies typically lead to widespread mortality because bleached corals cannot meet their energetic demands, and succumb to starvation, disease or overgrowth by algae and microbes^7^. Marine heatwaves of this type have become increasingly common in recent decades^3^, including the 2015–2016 El Niño, which triggered the worst global bleaching and mass mortality event on record^8,9^. Thus, although gradual ocean warming and acidification threaten the persistence of coral reefs over the next century^1^, marine heatwaves are already driving catastrophic coral loss across the world’s oceans^3,5^. Global climate models predict that these events will continue to increase in frequency^10^, such that bleaching is expected to occur every year for reefs in many parts of the world by mid-century^10,11^.

Diminishing intervals between recurrent heatwaves necessitate a shift in management focus from reef ecosystem recovery following such events to boosting coral survival through them, but the means of doing so remain unclear. Many conservation efforts to date have focused on measures to reduce the myriad of local reef stressors upon which climate change is superimposed, based on the premise that such efforts will enhance coral reef resilience (i.e., resistance and recovery) to heat stress^12^. However, empirical evidence for such effects is equivocal^12^. In fact, it has been argued that thermal tolerance may be correlated with tolerance to other stressors, such that mitigating local stressors might actually maladapt corals to future heatwaves^13^. Emerging alternative efforts to combat coral losses, such as assisted coral evolution, include attempts to leverage diversity in Symbiodiniaceae to facilitate rapid adaptive responses^14–18^. Yet, while manipulative experiments indicate that corals that change their symbionts during recovery from bleaching often become more heat tolerant as a result^15,19,20^, most of these studies have exposed corals to relatively short periods of thermal stress that may not reflect outcomes associated with the prolonged heatwaves that are anticipated under climate change^10^. Indeed, little is known about whether paradigms of coral bleaching developed from shorter events will hold true in future oceans. Moreover, with most of the world’s coral reefs now impacted by local anthropogenic disturbances, marine heatwaves—and their interaction with coral symbioses—must be considered in the context of multiple stressors.

Here, we investigated these interacting stressors on the world’s largest coral atoll, Kiritimati, where a tropical marine heatwave of unprecedented duration from 2015 to 2016 affected reefs ranging from near-pristine to ones highly impacted by chronic local human disturbance. We tagged individual colonies of two Indo-Pacific massive coral species (*Platygyra ryukyuensis* and *Favites pentagona*) at sites spanning this disturbance gradient, tracking symbiont identities and colony fates over seven expeditions that straddled this heatwave. Using ITS2 metabarcoding to identify symbionts and qPCR assays to quantify their abundances, we examined the influence of human disturbance on coral symbioses, and then quantified the timing of bleaching, recovery, and changes in symbiont community composition—testing for an effect of symbiont identity on coral bleaching and survivorship—throughout a prolonged and intense marine heatwave.

We found that, prior to the 2015–2016 heatwave, coral symbioses varied significantly with exposure to chronic local human disturbance: corals on highly disturbed reefs were typically dominated by heat-tolerant symbionts in the genus *Durusdinium*, while those on reefs exposed to lower levels of local disturbance tended to be dominated by heat-sensitive symbionts in the genus *Cladocopium*. Two months into the heatwave, corals dominated by heat-tolerant symbionts were less likely to have bleached compared to those dominated by heat-sensitive symbionts, as expected. But although the latter were quick to bleach, many of these coral colonies recovered from bleaching while still at elevated temperatures, a phenomenon that has not been observed previously. This recovery occurred through the proliferation of heat-tolerant *Durusdinium* symbionts. Furthermore, and contrary to previous work, corals dominated by heat-sensitive symbionts at the onset of the heatwave ultimately had higher (*Platygyra ryukyuensis*) or similar (*Favites pentagona*) survivorship through the heatwave compared to those that started with thermotolerant symbionts. This survival pathway–recovering from bleaching during a temperature anomaly–was only observed in corals at sites without very high levels of local anthropogenic disturbance. Our results demonstrate that corals have multiple pathways to survival through prolonged heatwaves—resistance and recovery—the relative importance of which depends on coral species, heatwave duration, and the prevalence of other anthropogenic stressors in the system.

## Results

### Chronic local human disturbance

**Fig. 1:**
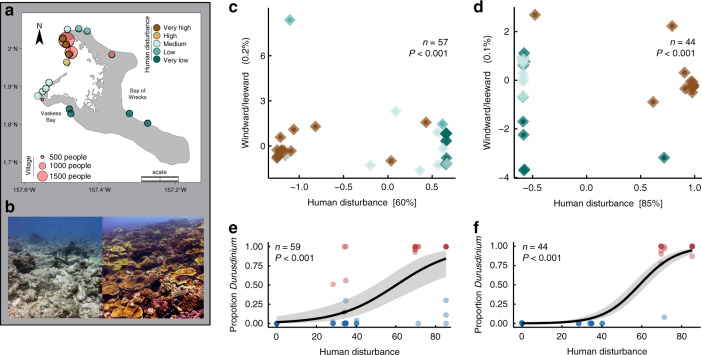
Chronic local human disturbance and coral symbiont assemblages. **a** Reef sites on Kiritimati (central equatorial Pacific) with tagged coral colonies, categorized by intensity of human disturbance. Entire scale bar shows 10 km. **b** Examples of Kiritimati’s reefs at very high (left) and very low (right) disturbance sites, prior to the 2015–2016 El Niño. For *Platygyra ryukyuensis* (**c**, **e**) and *Favites pentagona* (**d**, **f**): **c**, **d** Constrained ordination of amplicon sequence variants (ASVs) showing significant effects of human disturbance on symbiont communities, prior to the heat stress. Colors correspond to **a**. Values in square brackets show variation explained by each constrained axis; exposure (windward vs. leeward side of atoll) had no effect. **e**, **f** Logistic regressions showing the impact of human disturbance on the prevalence of *Durusdinium*. Blue dots indicate *Cladocopium*-dominated colonies, while red dots indicate *Durusdinium*-dominated colonies. Differences in sample size (**c**, **e**) reflect different sequencing depth thresholds used for analyses. Grey shading in **e**, **f** shows 95% CI. Sample sizes indicate individual coral colonies sampled before the heatwave.

Prior to heat stress, there was a strong signal of human disturbance on coral symbioses across Kiritimati’s reefs. Villages and human activities are located at one end of the atoll, while the rest of it remains largely unvisited by people, creating a gradient of chronic local human disturbance that we quantified based on population density and fishing pressure^21^ (Fig. 1a, see “Methods”). This gradient drives drastic variation in coral cover and reef complexity^22^ (Fig. 1b and Supplementary Fig. 1 and Supplementary Table 1), despite largely consistent oceanographic and thermal environments around the atoll^23^; reefs close to villages, for example, have similar average and extreme temperatures to those of less disturbed regions of the atoll (Supplementary Fig. 2 and Supplementary Table 2). We characterized the symbiont communities associated with 103 tagged colonies of two coral species (*P. ryukyuensis* and *F. pentagona*) at 12 sites along this disturbance gradient in 2014 and early 2015, using metabarcoding (Illumina MiSeq) of the commonly used internal transcribed spacer 2 (ITS2) region of ribosomal DNA^24^. We found that human disturbance significantly influenced the symbionts of both species (*P* < 0.001; Supplementary Table 3), and that this effect was stronger than that of any environmental variable (Fig. 1c–f, Supplementary Tables 4–6). While colonies at less disturbed sites were typically dominated by symbionts in the genus *Cladocopium*, colonies from areas exposed to high levels of human disturbance were dominated by the genus *Durusdinium* (Supplementary Fig. 3). These latter symbionts are generally considered to be stress-tolerant and have been shown to impart thermotolerance to many Indo-Pacific coral species^14,20^ (although other symbionts can also do this, e.g., in the Persian/Arabian Gulf^25^). This disturbance-dependence of symbiont identity may be the result of a combination of factors, including increased turbidity, sedimentation, and nutrient load^14^ (Supplementary Fig. 4), that may disadvantage *Cladocopium* relative to *Durusdinium*.

### Unprecedented heat stress and multiple stressors

**Fig. 2:**
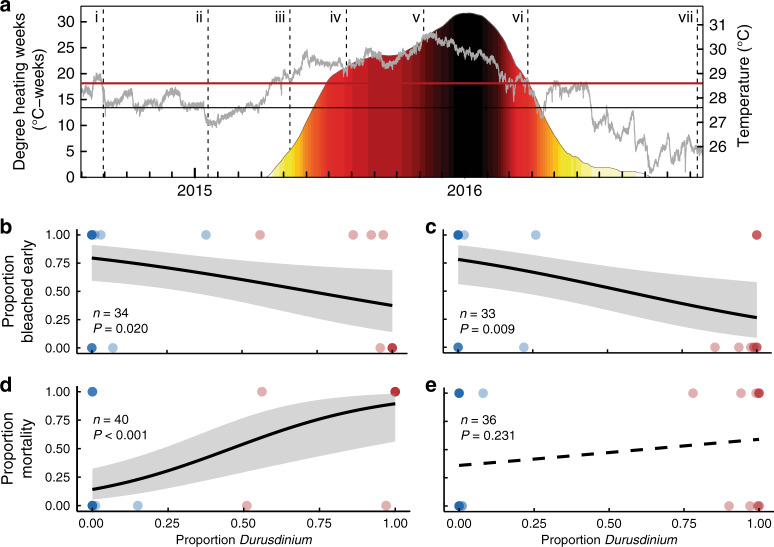
Thermal stress, and coral bleaching and mortality at the 2015–2016 El Niño’s epicenter. **a**, In situ temperature on Kiritimati (gray line), maximum monthly mean (black line) and bleaching threshold (red line; right axis). Shading shows cumulative heat stress (DHW; left axis) according to thermal thresholds: NOAA Coral Reef Watch Bleaching Alert 1 (yellow) and 2 (orange), “mass coral mortality” expected (red), and “not experienced by reefs”^29^ (maroon/black). Dashed vertical lines indicate the timing of expeditions. Tagged coral colonies could not be sampled in expedition v, but site-level photos were analyzed. **b–e** Logistic regressions for *Platygyra ryukyuensis* (**b**, **d**) and *Favites pentagona* (**c**, **e**), with data points for individual coral colonies dominated by *Cladocopium* (blue) or *Durusdinium* (red), showing the influence of symbiont composition (i.e., *Durusdinium*-dominance) on the propensity of colonies to have bleached two months into the El Niño (time point iv) (**b**, **c**), and the influence of symbiont composition before the heatwave (most recent sampling between time point i and iii) on colony survivorship through the end of the event (**d**, **e**). Intensity of blue and red shading denotes number of overlaid data points. Gray shading shows 95% CI. Sample sizes indicate individual tagged coral colonies.

During the 2015–2016 El Niño, a prolonged marine heatwave unfolded in the central equatorial Pacific with Kiritimati at its epicenter (Fig. 2a and Supplementary Fig. 2). Thermal anomalies on the atoll rapidly exceeded NOAA’s Coral Reef Watch (CRW) Bleaching Alert Level 1 and Alert Level 2 thresholds, and persisted for ten months, reaching an exceptional level of accumulated heat stress (31.6 °C-weeks; degree heating weeks (DHW); Fig. 2a) that was consistent around the atoll^23^ (Supplementary Fig. 2 and Supplementary Materials). This extreme heatwave was overlaid on Kiritimati’s spatial gradient of chronic local human disturbance (Fig. 1a), creating an ecosystem-scale natural factorial experiment that we leveraged to test the implications of symbiont identity and chronic human disturbance on coral bleaching and survival.

### Coral symbioses and bleaching resistance

We tracked the fate of tagged *P. ryukyuensis* and *F. pentagona* coral colonies throughout this heatwave, characterizing their symbiont assemblages (*n* = 363 samples from a total of *n* = 141 tagged coral colonies; mean = 2.6 time points per colony), and monitoring bleaching and survivorship. Corals of both species that were dominated by symbionts from the genus *Durusdinium* were significantly less likely to have bleached after two months of heat stress (and some may not have bleached at all; see Supplementary Discussion), compared to those dominated by *Cladocopium* (Fig. 2b, c; Supplementary Table 7). This corroborates past work that has demonstrated increased bleaching resistance in *Durusdinium-*dominated corals^14,15,20,26^.

### Coral survival

Contrary to previous findings from moderate heat-stress events^27,28^, however, corals with thermotolerant symbionts did not have higher survivorship by the end of the prolonged heatwave (Fig. 2d, e). Instead, we found that *P. ryukyuensis* colonies hosting *Durusdinium* eventually experienced dramatically lower survival (only 25%) compared to those initially dominated by *Cladocopium* (82%) (Fig. 2d and Table 1). Although there was a similar tendency for *F. pentagona* colonies hosting *Durusdinium* to have lower survival than conspecifics hosting *Cladocopium*, this pattern was non-significant (Fig. 2e, Table 1). These colony-level findings were further supported by benthic community composition data, which indicated that *P. ryukyuensis* underwent greater declines at very high disturbance sites compared to sites exposed to less local disturbance, while *F. pentagona* showed no clear pattern of differential change across the disturbance gradient (Supplementary Table 1).

**Table 1:** Coral symbionts and survivorship.

Symbiodiniaceae genus	*Cladocopium*			*Durusdinium*			Overall	
Survival status	Surv.	Died	Unk.	Surv.	Died	Unk.	Surv.	Died
Coral Species								
* P. ryukyuensis* (*n* = 59)	23 (82%)	5 (18%)	11	3 (25%)	9 (75%)	8	26 (65%)	14 (35%)
* F. pentagona* (*n* = 44)	14 (61%)	9 (39%)	4	6 (46%)	7 (54%)	4	20 (56%)	16 (44%)
Total across species (*n* = 103)	37 (73%)	14 (27%)	15	9 (36%)	16 (64%)	12	46 (61%)	30 (39%)

Number of colonies by outcome (survived (Surv.), died, unknown outcome (Unk.; e.g., lost colony)) late in the El Niño heatwave (March 2016), classified by starting dominant Symbiodiniaceae genus (>50% of reads). Sample sizes of colonies that were tracked, but had an unknown starting genus, because the initial expedition in which they were tagged was after the start of the heat stress (e.g., July 2015 or March 2016), are not included in this table, but are as follows: *P. ryukyuensis, n* = 22; *F. pentagona, n* = 16. Percentages represent percent of colonies within each starting Symbiodiniaceae genus that were known to either survive or die (i.e., Unk. colonies are not included in these percentages).

### Timing of recovery

**Fig. 3:**
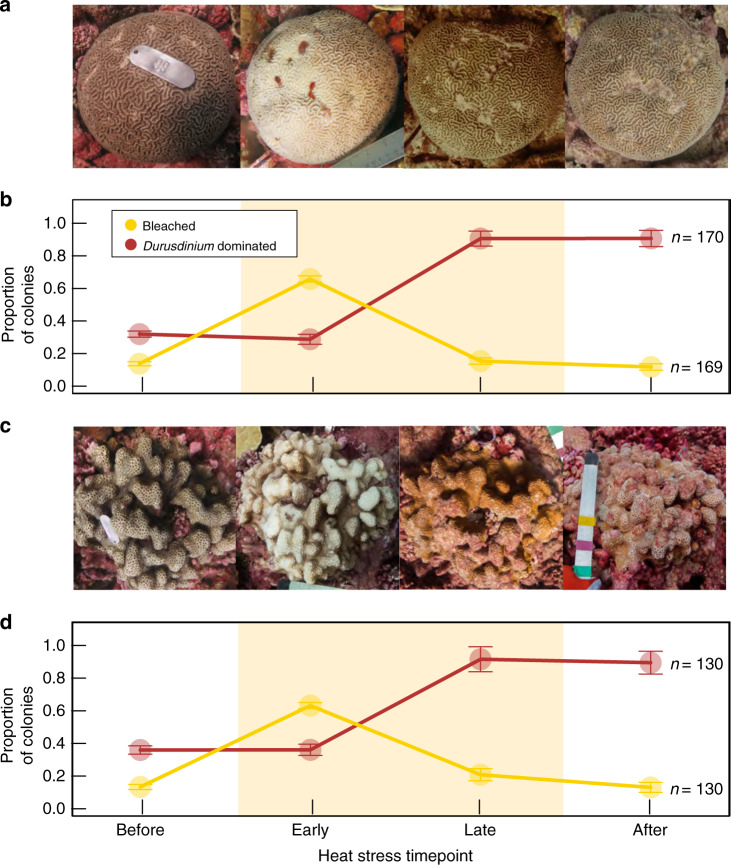
Progression (healthy–bleached–recovered) of coral colonies during the 2015–2016 El Niño. **a**, **c** Examples of single tracked coral colonies of **a** *P. ryukyuensis* and **c** *Favites pentagona* that bleached and recovered during the heatwave. **b**, **d** Proportion of coral colonies of **b** *P. ryukyuensis* and **d** *F. pentagona* that were dominated by heat-tolerant symbionts (>50% *Durusdinium* sequence reads) and proportion that were bleached (mean ± 95% CI), at time points spanning from before to after the El Niño-induced heat stress: before = the most recent time point before the heatwave (i–iii), early (iv), late (vi), and the most recent time point after the event (vii, viii). Expedition time points correspond to those in Figs. 2 and 4. Sample sizes refer to total number of samples (bleaching status or symbiont identities) taken from coral colonies across all time points. Shaded pale yellow areas in **b** and **d** correspond to >4 degree heating weeks from Fig. 2.

**Fig. 4:**
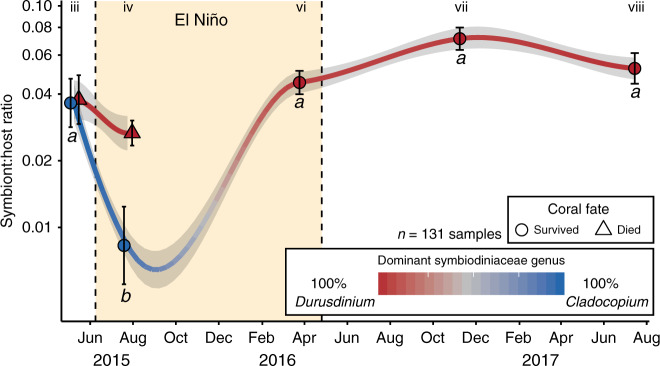
Changes in Symbiodiniaceae abundance and identity during the 2015–2016 El Niño. Mean symbiont:host cell ratios (±s.e.) at time points spanning from before to >1 year after the El Niño event for tracked *Platygyra ryukyuensis* coral colonies that survived (circles) or died (triangles). Sample size refers to the total number of measurements across all time points. Bleaching is indicated by a low cell ratio density (e.g., 0.01). Fitted lines show potential trajectories between sampled time points, and color (blue vs. red) indicates dominant Symbiodiniaceae genus in tracked colonies. The shaded pale yellow area between dashed vertical lines corresponds to >4 degree heating weeks from Fig. 2. Letters indicate significant differences between means for colonies that survived. Symbiont:host ratios were not significantly different across the two 2015 expeditions for colonies that died.

When not also exposed to very high levels of local disturbance, many of the corals that survived the heatwave recovered from bleaching while still at elevated temperatures (Fig. 3). This discovery challenges the current paradigm of coral bleaching, which holds that temperatures must return to normal before corals can recover and regain their symbionts^29–32^. We found colonies of both *P. ryukyuensis* and *F. pentagona* that had regained their pigmentation prior to our sampling late in the heatwave (Fig. 3 and Supplementary Fig. 5 and Supplementary Table 8), while still exposed to temperatures exceeding the bleaching threshold (Fig. 2a vi and Supplementary Fig. 2f). In both species, this recovery was associated with an increase in *Durusdinium-*dominated colonies (Fig. 3b, d). We corroborated this visual evidence of recovery by developing qPCR assays to quantify changes in overall algal symbiont abundance and dominant symbiont identity in tagged colonies of our best-sampled species, *P. ryukyuensis* (Fig. 4 and Supplementary Figs. 6 and 7). *Cladocopium*-dominated corals bleached quickly but then recovered at elevated temperatures: they had a baseline symbiont-to-host (S:H) cell ratio of 0.037 ± 0.010 (mean ± s.e.; time point iii), which dropped significantly (0.009 ± 0.003; linear mixed effects model: F = 18, *P* < 0.001) when bleached (time point iv), then returned to pre-bleaching levels (0.045 ± 0.006) by late-March 2016 (time point vi), and remained stable for the year following the heatwave (Fig. 4). It is likely that recovery of symbionts in this species occurred several months prior to our sampling in late-March 2016, because photos taken in November 2015 showed low levels of bleaching (Supplementary Fig. 8), and recovery to pre-bleaching symbiont levels typically takes months when bleaching is severe (e.g., refs. ^15,33^).

### Shifts in coral symbiont communities

**Fig. 5:**
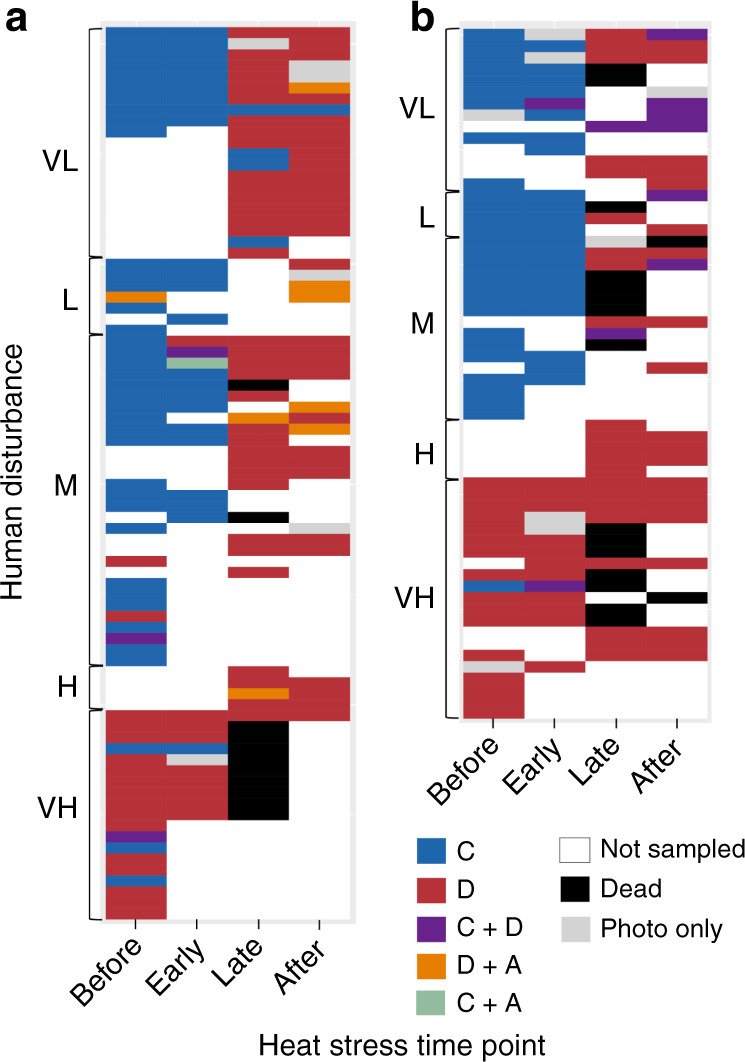
Time series of dominant symbiont genera in each tagged coral. **a** *Platygyra ryukyuensis* (*n* = 81). **b** *Favites pentagona* (*n* = 60). Cell color indicates dominant symbiont genus (former clades: C *Cladocopiu*m; D *Durusdinium*, A *Symbiodinium*). Co-dominance is shown if the second most-abundant genus was >20% of the total sequence reads. Time points from Fig. 2 are grouped as follows (Before: time points i–iii; Early: time point iv; Late: time point vi; After: time point vii or later).

Recovery of corals at elevated temperatures was driven by the proliferation of the thermally tolerant symbiont *Durusdinium*, often resulting in complete shifts in symbiont communities (Figs. 4 and 5). *Durusdinium* ITS2 profiles, which were extremely rare in colonies at less disturbed sites (very low to medium category) before the heatwave (*P. ryukyuensis*: 8.96 ± 3.89%; *F. pentagona*: 0.07 ± 0.01%, mean ± s.e.), increased enormously during their recovery (*P. ryukyuensis*: 90.42 ± 3.34 %; *F. pentagona*: 83.07 ± 5.30%, mean ± s.e.) (Supplementary Fig. 3). Thus, contrary to work concluding that rare symbionts are transient^34^ and have little functional significance^35^, our results support studies that suggest that rare heat-tolerant symbionts may be essential to coral recovery from heat stress^26,36^ and potentially of profound functional significance for long-term coral persistence.

## Discussion

This natural experiment highlights that, rather than being uniform for any given reef, both bleaching and recovery thresholds vary across different coral-symbiont associations, and this variability can give rise to alternate pathways to coral survival through extended heatwaves. Specifically, *Durusdinium*, which has been shown to increase the thermal tolerance of corals by at least 1–1.5 °C^20,36,37^, was linked to bleaching resistance in this study, with *Durusdinium*-dominated colonies of both species less likely to bleach initially during intense heat stress. This bleaching resistance pathway to survival represents the dominant model of how some corals might survive future warming^16,38,39^. However, to the contrary, we found that colonies with bleaching-resistant symbionts actually did not have a survival advantage over those with heat-sensitive symbionts. Here, we argue that symbiont-mediated variation in bleaching thresholds creates a recovery pathway to survival in which corals with heat-sensitive symbionts may bleach first as temperatures rise, but then subsequently recover with thermotolerant symbionts at temperatures which, while still elevated, are below the latter symbiont’s bleaching threshold. The existence of this alternative recovery pathway to survival is a logical extension of theory on bleaching resistance, but the possibility of recovery at elevated temperatures has been debated^7,31,40,41^ and never before been documented, presumably because previously studied heatwaves have never persisted long enough to observe this phenomenon.

Distinct survival pathways were strongly tied to chronic anthropogenic disturbance, and recovery—not resistance—was the predominant means of coral survival through the heatwave. Of the surviving colonies that we had also sampled two months into the heatwave, approximately two-thirds had already bleached, demonstrating the importance of recovery over resistance in driving resilience. We suggest that, because thermotolerant *Durusdinium* contribute less energy to their coral hosts^42^, the differences in eventual survivorship are the result of variation in energy reserves^42^, homeostatic function or immune capacity^43,44^, exacerbated by environmental conditions at highly disturbed sites (such as increased physical disturbance, dissolved organic carbon and microbial activity^45^) which further reduce the ability of corals to survive extended heatwaves. Thus, human disturbance appears to offer a trade-off to at least some corals, with heat-tolerant symbionts offering short term bleaching resistance but reducing their capacity for survival in the event of prolonged heat stress. Although this trade-off is not universal, even among the two species studied here, it implies that local disturbance and coral survival in the face of climate change can be tightly linked.

Our study has important implications both for managing corals and predicting their responses to future climate change. By linking local disturbance with coral persistence via fitness trade-offs associated with hosting different symbionts, this study sets the stage for manipulative experiments that can uncover the mechanisms underlying the relative success of alternate survival pathways. Testing how different coral host-symbiont combinations and disturbance conditions affect these pathways will allow us to better manage coral reefs through future prolonged heatwaves. With heatwaves predicted to increase in duration this century, we expect that the recovery pathway, which may offer a fitness advantage to some species over initially possessing thermotolerant symbionts, may become an increasingly important means of coral survival. Accordingly, climate models that assume uniform bleaching and recovery thresholds, instead of explicitly incorporating multiple thresholds, may be inaccurate. Reframing the current paradigm of coral bleaching and integrating it with new theory about coral symbioses under multiple stressors, could greatly improve our ability to forecast future reef states and employ targeted interventions to enhance coral survival. Persistence of coral reef ecosystems inarguably depends primarily on large-scale mitigation of greenhouse gas emissions^46^, but the ecological surprise detected in this study provides a glimmer of hope for some corals even under extreme climate scenarios.

## Methods

### Study location

Kiritimati (Christmas Island), Republic of Kiribati, is located in the central equatorial Pacific Ocean (01°52′N 157°24′W), at the center of the Niño 3.4 region (a delineation used to quantify El Niño presence and strength^47^). Kiritimati is the world’s largest atoll by landmass (388 km^2^; 150 km in perimeter), and all fifteen surveyed reefs surrounding the atoll are sloping, fringing reefs with no back reef or significant reef crest formations. Kiritimati has a strong spatial gradient of human disturbance around the island, with the majority of the human population residing in two villages on the northwest side of the atoll (Fig. 1a)^48,49^. Human uses, including waste-water runoff, subsistence fishing, and a large pier, are densely concentrated in this area, while the north, east, and south regions of the atoll are minimally impacted^48,49^. We quantified the intensity of chronic local human disturbance at each site, using two spatial data sources, human population densities, and fishing pressure (Supplementary Table 9). First, as a proxy for immediate point-source inputs from villages into the marine environment such as pollution and sewage runoff, we generated a geographic buffer (in ArcGIS) to determine human population size within 2 km of each site. Nearly all individuals live in villages, and village location was mapped based on published field surveys^49^. Population size for each village was extracted from the 2015 Population and Housing Census from the Kiribati National Statistics Office^50^. Secondly, to account for the more diffuse effects of subsistence fishing on the reef ecosystem, we generated a kernel density function with ten steps based on mapped fishing intensity from the work of Watson et al.^49^. We weighted each metric equally, and from this combined metric we grouped sites into five distinct disturbance categories, termed very low, low, medium, high, and very high for use in Fig. 1a, c, d (Supplementary Fig. 1 and Supplementary Table 9, refs. ^48,49^). In all other statistical analyses, we treated human disturbance as a continuous variable, and square root transformed the combined metric to account for the large gap in values between medium/high and very high sites.

To rule out potentially confounding variables resulting from the spatial clustering of human disturbance, we quantified multiple oceanographic and abiotic variables at each site (Supplementary Table 2) and tested for an effect of each variable on symbiont composition by conducting logistic regressions (Supplementary Methods). We defined site exposure (i.e., windward versus leeward) based on the dominant wind direction (southeasterly;^51^), measured salinity, pH, and dissolved oxygen (DO) saturation in situ, and extracted remotely-sensed wave energy, and maximum and mean net primary productivity for each site from the open source data product Marine Socio-Environmental Covariates (MSEC; https://shiny.sesync.org/apps/msec/^52^). We then fit linear models of each parameter (See Supplementary Methods for an in-depth overview of methodologies for different parameters). We also tested for relationships between human disturbance and indicators of sedimentation, turbidity, and bacterial loads (See Supplementary Methods and Supplementary Fig. 4).

### Temperature quantification

We deployed temperature loggers (SBE 56, Sea-Bird Scientific) around the atoll at a subset of our study sites (all between 10 and 12 m depth; at least one logger deployed in each disturbance treatment) from 2011 to 2016, to measure in situ thermal stress (Supplementary Fig. 2 and Supplementary Methods). Corals are sensitive to temperatures warmer than 1 °C above their maximum monthly mean sea surface temperature (SST), defined as the bleaching threshold^53^. We calculated local bleaching thresholds using in situ data to offset satellite measurements in 6 distinct regions around the atoll (Supplementary Fig. 2) as in Claar et al.^23^, this allowed us to ensure that bleaching thresholds were determined from long-term temperature records. We then used in situ temperature data and local bleaching thresholds to calculate degree heating weeks^54^ for each region. As temperature profiles were similar among sites^23^ (Supplementary Fig. 2 and Supplementary Table 10), and not all sites had temperature data for the complete time period, we also averaged temperature and bleaching thresholds across regions to produce a measure of island-wide temperature and thermal stress (degree heating weeks, DHW). We further compared our in situ DHW values to the NOAA 5-km satellite product, yielding consistent results (see Supplementary Methods and Results and Supplementary Fig. 2).

### Coral tagging and sampling

We tagged and sampled coral colonies of two species (*n* = 141 total; Fig. 4) along a 60 m transect, laid along the 10-12 m isobath, at each of 15 different fore reef sites around Kiritimati (Fig. 1a), during expeditions before (August 2014, January/February 2015, April/May 2015), during (July 2015, March 2016), and after (November 2016, July 2017) the El Niño heatwave. Two sites (H1, VL4) were sampled for the first time in March 2016 and one site was sampled for the first time in July 2015 (VL2). These sites were therefore not included in analyses presented in Fig. 1c–f. Sample sizes by site are presented in Supplementary Table 11. Each coral colony was photographed at its initial tagging and at each revisit to record colony measurements and bleaching (see Supplementary Methods). Not all sites could be visited during all field seasons, and some site surveys were only partially completed during some field seasons due to inclement weather conditions. Because the coral mortality event was associated with broad transformation and degradation of the reefs of Kiritimati^22^, some colonies that were presumed dead had disappeared between July 2015 and March 2016. We tested the sensitivity of our results to different assumptions about coral mortality (see Supplementary Methods) and found that the significance statuses of mortality analyses were robust to the set of assumptions used (Supplementary Table 12).

We sampled corals using a small chisel and stored the small tissue samples extracted in seawater on ice until preservation. After collection, we preserved one portion of each coral tissue sample in guanidinium buffer (50% w/v guanidinium isothiocyanate; 50 mM Tris pH 7.6; 10 µM EDTA; 4.2% w/v sarkosyl; 2.1% v/v-mercaptoethanol) which we stored at 4 °C until extraction for sequencing. We froze a second portion of each sample at −20 °C in the field, and subsequently stored these samples at −80 °C until DNA extraction.

### Coral cover quantification

At each of the fifteen sites, we photographed the benthic community underneath a 1 m^2^ gridded quadrat that was randomly placed on the substrate, at twenty-four to thirty points along the 60 m transect (*n* = 1389 photos total). Each site was sampled before the bleaching event (e.g., July 2013, August 2014, January 2015, May 2015) and after the bleaching event (e.g., November 2016, July 2017). Two Medium disturbance sites were also sampled in November 2015 to assess bleaching during the El Niño. Tagged coral colonies were also located along these transects at each site. We analyzed all photos using CoralNet beta version^55^, an online software for benthic image analysis, by projecting 100 random points onto each image and manually annotating the substrate beneath each point. Percent cover estimates for each site were calculated by averaging across all photos from that site within each time point (Supplementary Table 1). We further used a haphazard sampling of photos taken from medium and very high disturbance sites to corroborate November colony bleaching observations from CoralNet with a greater sample size (see Supplementary Methods).

### DNA extraction

For the samples prepared for Illumina MiSeq amplicon sequencing, we performed DNA extraction using a guanidinium-based extraction protocol^26,56^ with the modification that the DNA pellet was washed with 70% ethanol three times rather than once. After extraction, we cleaned the DNA using Zymo Genomic DNA Clean and Concentrator^TM^-25 (Catalog Nos. D4064 & D4065) following the standard protocol (http://www.zymoresearch.com/downloads/dl/file/id/638/d4064i.pdf). We quantified DNA using the dsDNA Qubit assay. For the samples prepared for qPCR, we placed a portion of the frozen sample in 1% SDS in DNAB and extracted these sub-samples using an organic extraction protocol for qPCR assays^57^.

### High-throughput (MiSeq) amplicon sequencing

We used amplicon sequencing as the primary means of characterizing the symbiont assemblages associated with each coral. We chose the ITS2 amplicon for high-throughput sequencing because it is currently the standard region used for identification and quantification of Symbiodiniaceae taxa. ITS2 is phylogenetically^58^, functionally and ecologically^59^ informative. Library preparation for Illumina MiSeq ITS2 amplicon sequencing was performed by the HIMB Genetics Core Lab following the Illumina 16S Metagenomic Sequencing Library Preparation (Illumina protocol, Part # 15044223 Rev. B) with the following modifications:

- ITS2 primers (ITSD-forward: 5’-TCG TCG GCA GCG TCA GAT GTG TAT AAG AGA CAG GTG AAT TGC AGA ACT CCG TG-3’^60^ and ITS2-reverse: 5’-GTC TCG TGG GCT CGG AGA TGT GTA TAA GAG ACA GCC TCC GCT TAC TTA TAT GCT T-3’^61^) were used in place of the 16S primers.
- PCR 1 annealing temperature was 52 °C, PCR 1 was performed in triplicate, and PCR product was pooled prior to bead clean.
- A 1:1.1 ratio of PCR product to SPRI beads was used for PCR 1 and PCR 2 clean up.

Samples were sequenced on the Illumina MiSeq platform with 2 × 300 bp paired-end read chemistry. We attempted to sequence all samples taken from tagged corals (and preserved in guanidinium buffer^26,56^) but some samples failed to sequence and were therefore not included in the study. The total number of coral samples successfully sequenced was 363 (*P. ryukyuensis*, *n* = 210; *F. pentagona*, *n* = 153).

### Bioinformatics

Quality filtering, sequence assembly and taxonomic assignment were performed using the dada2 pipeline (v1.12.1) for amplicon sequence variants (ASVs) and the SymPortal framework for symbiont taxa^62^. ASVs were filtered and analyzed using the dada2 package in R and then re-formatted for input into the R package phyloseq^63^ (v1.28.0). We further filtered the phyloseq object to remove samples with low sequence abundances due to amplification issues. For ASV analyses, we removed samples with less than 1000 reads.

SymPortal controls for intragenomic variation (e.g., ref. ^64^) by identifying putative Symbiodiniaceae taxa (referred to as ITS2-type profiles) based on the idea that a specific group of ITS2 sequences always present together represents a single Symbiodiniaceae genotype. The SymPortal framework (v0.3.18) utilizes the mothur v. 1.39.5 pipeline^65^ with additional executables from the BLAST+ suite^66^, minimum entropy decomposition^67^ and custom python functions to run quality control and assembly of ITS2 sequences. These sequences are then loaded into the SymPortal database where co-occurring sets of ITS2 sequences (called defining intragenomic variants; DIVs) are compared with previously imported data to identify what are referred to as ITS2-type profiles. Final output from the SymPortal framework for the present study consisted of sequence abundances of each ITS2-type profile for every coral sample. All analyses conducted at the genus level utilized this output by summing across ITS2 profile sequence counts for each genus. For analyses on ITS2-type profiles, we removed all samples with less than 250 sequence reads. Note that 95% of samples had greater than 1000 sequence reads.

### Measuring symbiont to host cell ratios using quantitative-PCR assays

We used quantitative PCR assays targeting specific loci in *P. ryukyuensis*, *Cladocopium*, and *Durusdinium* to measure symbiont to host cell ratios^68^ (*n* = 57 colonies; *n* = 146 samples), a measure of symbiont density, during the period of May 2015 and July 2017. We attempted to include all 60 (out of 81) colonies that were sampled during this time period but were unable to sequence three due to lack of remaining sample. For the analysis in Fig. 4, only corals with known outcome (survived/died) were included (*n* = 48 colonies; *n* = 131 samples). Assays targeting actin loci specific to *Cladocopium* and *Durusdinium* were multiplexed following the methods of^69^ in 10 µL reactions. We designed a new assay targeting the PaxC intron of *P. ryukyuensis* (which is single copy in corals^70^) based on 14 sequences obtained from NCBI Nucleotide database (accession numbers KX026897–KX026910;^71^). Forward and reverse primers (PaxC-F: 5′-GGATACCCGCGTCGACTCT-3′; PaxC-R: 5′-CCCTAAGTTTGCTTTTTATTGTTCCT-3′) were designed using Primer Express v3.0 (Applied Biosystems) to amplify a 72 bp region of the PaxC intron. We performed amplification of the coral host target locus in 12.5 µL qPCR reactions containing 900 nM of each primer using SYBR Green Chemistry (Power SYBR Green MasterMix, Applied Biosystems). The amplification efficiency of this assay was measured as 99.27% using a 4-log10 dilution series of *P. ryukyuensis* DNA.

We assayed DNA extracted from each sample in duplicate with the *P. ryukyuensis* PaxC assay and with the multiplexed *Cladocopium* and *Durusdinium* assays on a StepOnePlus qPCR platform (Applied Biosystems). Thermal cycling consisted of initial incubation at 50 °C for 2 min and 95 °C for 10 min, followed by 40 cycles of denaturation at 95 °C for 10 s and annealing/extension at 60 °C for 1 min. Cycle threshold (Ct) values (with the fluorescence threshold set to 0.01) for each target were adjusted to account for differences in fluorescence intensity among assays based on the amplification of copy number standards (following the methods of ref. ^69^). We then calculated symbiont to host cell ratios using the formula 2^(Ct(host) − Ct(symbiont)), and adjusting to account for (1) differences in target locus copy number (see below), (2) differences in symbiont and host ploidy^72^, and (3) differences in DNA extraction efficiency^69^. We calculated symbiont to host cell ratios using R code available at github.com/jrcunning/steponeR.

### Determination of actin copy number in *Cladocopium* and *Durusdinium* in *P. ryukyuensis*

We extracted *Cladocopium and Durusdinium* cells from frozen tissue (three samples *Cladocopium*, three samples *Durusdinium*), counted the number of Symbiodiniaceae cells under a microscope using a hemocytometer, and extracted DNA from three replicate aliquots of 20,000 cells from each sample. DNA template equivalent to 2000 cells from each extraction was then assayed along with copy number standards. We generated copy number standards (e.g., known numbers of copies of each qPCR target locus) by the purification of each amplified target (Wizard SV Gel and PCR Clean-up System, Promega), quantified nucleic acid concentration using a Nanodrop-1000, and converted to number of copies based on the length of the amplicon and a conversion factor of 660 Daltons per base pair. We used a log2 dilution series from 64,000 copies to 2000 copies of *Cladocopium and Durusdinium* targets as a standard curve to quantify the number of copies present in DNA extracted from known numbers of Symbiodiniaceae cells.

### Statistical analysis

To assess the factors driving differences among Symbiodiniaceae communities, we first performed a set of canonical analyses of principal coordinates (CAP) using ASV data. CAP is a constrained ordination method, which allows for direct comparison of environmental variables and changes in Symbiodiniaceae community composition by constraining ordination axes to linear combinations of the environmental variables. After exhausting all potential constrained axes, residual variability is addressed by fitting additional unconstrained axes (which represent linear variability which is caused by factors not included in the constrained axes). We constructed a Symbiodiniaceae phylogenetic tree for use in this ordination by aligning sequences from each genus separately using AlignSeqs from DECIPHER (v2.12.0)^73^ and wrote the trees using write.tree from ape (v5.3)^74^. After we aligned sequences within each genus, we created a distance matrix using nr28s-rDNA distances (divergence of the D1–D3 region of the 28S;^75,76^) to describe between-genera distances. Using upgma (R package phangorn^77^ v2.5.5), we then created a phylogenetic tree and imported it into the phyloseq object before statistical analysis. We conducted ordination with the function ordinate (phyloseq^63^), using weighted unifrac distances, and included exposure and local human disturbance level (very high, high, medium, low, and very low). After ordination, we conducted an ANOVA-like permutation test to determine if the defined model was significant (anova.cca, vegan package^78^ v2.5-5). We confirmed these results using an automatic stepwise model building tool to build and evaluate the significance of constrained axes using permutation p values (ordistep tool, R package vegan^78^). Full model outputs are presented in Supplementary Table 5. We also computed the variance inflation factors (vif.cca, R package vegan^78^) to test for redundant constraints, or for multicollinearity between factor levels. All variance inflation factors were relatively small (<2.5), so multicollinearity is unlikely to influence our results.

Three sets of logistic regressions were performed on genus-level data (derived from ITS2 profiles). First, the proportion of sequence reads assigned to the genus *Durusdinium* (quasi-binomial data structure) was fit as a function of local disturbance, using all colonies tagged up to and including April/May 2015 (time points i–iii). For colonies sampled more than once during this period, the latest of the samplings within this period were used. Second, we tested for an effect of the proportion *Durusdinium* reads on the probability of bleaching in July 2015 using binomial bleaching data and sequence data both from that time point (iv). Third, we regressed survival status (binomial; alive or dead) against proportion *Durusdinium* reads from samples taken closest to the heatwave for each colony (time points i–iii). Logistic regressions were run in R using the “bayesglm” function (from package “arm” v1.10-1) which is more robust to low sample sizes. However, we compared these model outputs to a typical glm and these produced the same significance for all models.

Symbiont-to-host ratios for *P. ryukyuensis* were compared across time using linear mixed effects models with field season as a fixed effect and coral ID as a random effect using lmer (lme4 v1.1-21)^79^. Models were fit separately to colonies that survived versus died. A posthoc test with Tukey correction was conducted using emmeans^80^ (v1.3.5).

### Reporting summary

Further information on research design is available in the Nature Research Reporting Summary linked to this article.

## Supplementary information

Supplementary Information

Reporting Summary

## Data Availability

All data that support the results of this study are available on GitHub (https://github.com/baumlab/Claar_etal_2020_NatCom) with the following identifier: 10.5281/zenodo.4014057. We used previously collected data for the PaxC intron of P. ryukyuensis from NCBI Nucleotide database (accession numbers KX026897–KX026910) to develop qPCR probes. Next-generation sequencing data are available in the NCBI Sequence Read Archive Accession Number PRJNA543540.

## Code availability

All scripts required to replicate the analyses presented in this study are available on GitHub (https://github.com/baumlab/Claar_etal_2020_NatCom) with the following identifier: 10.5281/zenodo.4014057.
