# Supplementary material for: Dynamic symbioses reveal pathways to coral survival through prolonged heatwaves
Source: Nat Commun. 2020 Dec 8;11:6097. doi: 10.1038/s41467-020-19169-y (PMC7723047; doi:10.1038/s41467-020-19169-y)
Supplement: Supplementary file 1 — Supplementary Information [file 41467_2020_19169_MOESM1_ESM.pdf]

Supplementary Materials for Claar et al.

Dynamic symbioses reveal pathways to coral survival through prolonged heatwaves

Table of Contents

Supplementary Methods **........ 2**

Environmental parameters........ 2

Indicators of human disturbance........ 2

Temperature........ 3

In situ temperature measurements........ 3

Satellite thermal stress........ 4

Coral survivorship........ 5

Bleaching........ 5

Visual assessment of bleaching status........ 5

Assessing coral bleaching in November 2015........ 6

Supplementary Discussion........ 6

Bleaching resistance in Favites pentagona........ 6

Implications of temperature quantification methodology........ 7

Supplementary Tables........ 9

Supplementary Figures........ 22

Supplementary References........ 30

Supplementary Methods

Environmental parameters

We quantified multiple oceanographic and abiotic parameters at each site to assess if these factors influenced coral symbiont communities (Supplementary Table 2). We measured salinity, pH, and dissolved oxygen (DO) saturation at each site using a YSI Pro Plus handheld multiparameter meter that was calibrated daily. We also extracted remotely sensed wave energy, and maximum and mean net primary productivity for each site from the open source data product Marine Socio-Environmental Covariates (MSEC; https://shiny.sesync.org/apps/msec/ [1]). MSEC productivity values are calculated over a 2.5 arcmin grid based on data from NOAA CoastWatch, which models net primary productivity using satellite-derived measures of photosynthetically available radiation, SST, and chlorophyll *a* concentration. For wave energy, only sites that had available NOAA Wave Watch 3 data in the MSEC database were included in statistical analyses. To test whether any of these factors were potentially confounding the impacts of chronic human disturbance on symbiont communities, we constructed a series of quasibinomial logistic regression models with proportion *Durusdinium* as the response variable, including both human disturbance and each additional parameter as explanatory variables. We found no significant effect of any environmental parameter with these models (Supplementary Table 6).

Indicators of human disturbance

We tested whether our quantified metric of local human disturbance (quantified as the combined effects of population density and fishing pressure) was correlated with indicators of turbidity, sedimentation and microbial load. As a proxy for sedimentation, we used benthic quadrat photos (see Methods) to calculate an estimate of the percent of the substratum covered by sediment. We

then tested for a relationship between human disturbance and percent sediment cover using a linear model. We calculated this percent sediment metric both including and excluding sand and modelled each separately. As a proxy for turbidity, a single experienced scientific diver (K. Tietjen) estimated visibility at each dive site. In expeditions where a site was sampled on more than one day (up to $n = 3$), these estimates were averaged. We then averaged this visibility across all expeditions for which we had data for a given site. We tested for a relationship between visibility and human disturbance using a linear model. Finally, we evaluated published data[3] on the concentration of bacteria in the water column at four sites on Kiritimati (two very high and two very low human disturbance). These data were collected by taking water samples (1-2 mL), preserving them in formaldehyde and then filtering the samples and counting DAPI-stained bacteria under high magnification. The mean concentrations of microbes at each site ($n = 4$ samples each) were then compared using a one-way ANOVA and a Tukey post-hoc test (Supplementary Fig. 4d).

Temperature

In situ temperature measurements

Temperature was measured by deploying Sea-Bird 56 loggers at a minimum of one site within each region. Temperatures were averaged across sites within each region and standardized to 1-hr and 1-day sampling frequency; all associated methods and data are available in [1]. To demonstrate daily variability for each region (Supplementary Fig. 2b, c), hourly *in situ* temperature was used to calculate the temperature range for each day as (maximum temperature) - (minimum temperature) for before El Niño conditions (9-September-2014 to 9-June-2015) and during El Niño conditions (9-June-2015 to 26-April-2016). Daily temperature was also plotted

for each region for a time period before El Niño (September 2014), during El Niño (September 2015), and more broadly throughout the time series (1-February-2015 to 1-September-2016) (Supplementary Fig. 2d, e, f). Coral bleaching threshold (mean monthly maximum (MMM) temperature + 1°C), including an offset for *in situ* measurements[2], was also plotted for each region (Supplementary Fig. 2f), showing that most regions had similar bleaching threshold, with the exception of Bay of Wrecks which was slightly higher. A comparison of thermal stress between *in situ* and satellite-derived (NOAA) measurements is shown in Supplementary Table 9. This includes comparisons of satellite-derived thermal stress with *in situ* thermal stress calculated based on the offset MMM (described above), as well as *in situ* thermal stress calculated based on NOAA's satellite-derived MMM for Kiritimati. We chose to include *in situ* thermal stress calculated based on the offset MMM in the main text, as we believe that it provides the most accurate picture of local warming conditions since it includes locally derived bleaching thresholds and local, reef-depth temperature measurements.

Satellite thermal stress

NOAA's CRW Daily Global 5-km Satellite Coral Bleaching Heat Stress Degree Heating Week version 3.1 product ([4]; Supplementary Fig. 2g) shows similar temperature trajectories as our *in situ* measurements[2]. However, *in situ* thermal stress (°C-weeks), measured with the mean monthly maximum (MMM) offsets calculated in [2], was consistently higher than both satellite-derived thermal stress and *in situ* thermal stress calculated without local MMM offsets. The satellite-derived maximum heat stress (extracted from NOAA's 5-km Coral Reef Watch product; measured as °C-weeks) for each disturbance treatment varied by less than 1°C-week (~4%

overall; [2]). To demonstrate that Kiritimati was located at the epicenter of the 2015/2016 El Niño, the map in Supplementary Fig. 2h was created using the thermal stress data calculated in [5].

Coral survivorship

When tags were relocated, corals were either assigned as “alive” (at least one polyp still present and visibly alive), “dead” (skeleton present but no live polyps) or “gone” (colony presumed dead but entire skeleton has eroded, or no longer in same location; i.e., colony had dislodged). We assumed that a colony assigned to “gone” was killed by the bleaching event only if it had been seen in the previous expedition (i.e., July 2015), otherwise it was excluded. However, to test the sensitivity of our analyses to this assumption, we performed analyses involving mortality using two other sets of criteria: 1) only corals recorded as “dead” were considered to have died, “gone” colonies were excluded; 2) all corals recorded as “gone” were considered to have died. The significance statuses of mortality analyses were found to be robust to these widely varying assumptions (Supplementary Table 13).

Bleaching

Visual assessment of bleaching status

Photographs were taken of each tagged colony with a scale at each time point and used to assess bleaching status. We assigned colonies to one of four bleaching severity groups based on visual criteria: 1) no bleaching or paling; 2) some light bleaching but less than 5 cm across largest patch and less than 50% of colony pale; 3) bleaching in patches >5 cm or more than 50% of colony pale; 4) bleaching and/or paling >80% of colony. Recovery was considered to have occurred between July 2015 and March 2016, if the bleaching status of a colony improved (i.e.,

decreased). For binomial treatments of bleaching, we considered categories 1 and 2 to be "healthy" and categories 3 and 4 to be "bleached". Thus, colonies were assigned to "bleached" if they had at least one patch of their surface that was bleached and greater than 5 cm across, or if more than 50% of the surface of the coral was faded.

Assessing coral bleaching in November 2015

Midway through the heatwave, in November 2015, we were unable to sample tagged coral colonies (due to funding constraints that prevented our primary team from conducting an expedition to Kiritimati), but a separate team conducted benthic photo quadrats at four of our monitoring sites (two medium and two very high disturbance sites) as well as a set of landscape images at these sites. We analyzed both types of photos and quantified the bleaching status (bleaching or healthy; see above) of all *Platygyra ryukyuensis* and *Favites pentagona* colonies visible in any of these photos. This included CoralNet analyses of the benthic photo quadrats (described in Methods), as well as assessment of individual colonies in the landscape images (where resolution was high enough to evaluate bleaching of individual colonies) and in colonies that were peripheral to the benthic quadrats but still captured in the quadrat images.

Supplementary Discussion

Bleaching resistance in Favites pentagona

Several *Durusdinium*-dominated *Favites pentagona* colonies from very high disturbance sites were not bleached at either of the time points we sampled tagged colonies during the heatwave (July 2015 or March 2016). To provide some indication of whether these colonies may have bleached and recovered between these sampling time points, or whether they were likely

resistant to bleaching throughout the duration of the event, we assessed differences in partial mortality of *F. pentagona* colonies. The logic here was that almost all of the *Cladocopium*-dominated colonies of this species that we know bleached severely experienced substantial partial mortality (89% experienced greater than 10% mortality; Supplementary Table 14), suggesting a link between bleaching and partial mortality in this species. We found that *Durusdinium*-dominated *F. pentagona* colonies showed very little partial mortality, with all colonies showing less than 10% colony loss. This suggests that these colonies did not bleach severely during the heatwave, as we would have expected more partial mortality to accompany this bleaching.

Implications of temperature quantification methodology

One major conclusion of our study is that at least some corals are able to recover from bleaching while still above the bleaching threshold of their initial symbiont community. We showed this by sampling tagged corals that were bleached in July 2015 and healthy in March 2016, and further demonstrated that both of these time points were above the mean monthly maximum for all reefs (Supplementary Fig. 2f). Our estimates of region-level bleaching threshold came from using atoll-wide satellite temperature data with regional offsets calculated from 2-4.5 years of *in situ* data depending on region (see Claar et al[2]). This analysis therefore relies on the assumption that temperature patterns over the previous 4.5 years are representative of the true differences in temperatures that have existed across regions over the timescale relevant to establishing coral bleaching thresholds. While we have no reason to suspect that the 4.5-year period used to calculate offsets was distinct from the longer temperature history of Kiritimati (at least at the scale relevant to establishing bleaching thresholds), we note that our main conclusion does not

hinge on this assumption. If we instead use the NOAA mean monthly maximum (28.0°C; without offsets) to calculate an atoll-wide bleaching threshold of 29.0°C (which does not incorporate the local upwelling captured by our in situ data[1]), we see that in March 2016, sites in the very low disturbance region Bay of Wrecks were still above even this higher threshold. At these sites alone, we observed 5 colonies (*P. ryukyuensis*, $n = 2$; *F. pentagona*, $n = 3$) that had recovered while still above the threshold. Thus, this important conclusion is robust against changes in the resolution at which we estimate the bleaching thresholds of corals on a given reef.

Supplementary Tables

Supplementary Table 1 | Change in coral cover over the El Niño-induced heatwave. Overall coral cover before the heatwave and percent change in cover of *Platygyra ryukyuensis* and *Favites pentagona* (NA= percent cover was <0.1%). Sites H1, M5, L2, and VL4 are not included, because before El Niño cover was unavailable for those sites.

**Site Name**	**Site ID**	**Disturbance Category**	**Overall coral cover**	***Platygyra ryukyuensis***	***Favites pentagona***
			**Before (%)**	**Change (%)**	**Change (%)**
VH1	27	*very high*	1.7	-71.3	NA
VH2	32	*very high*	3.5	-100	-62.6
VH3	30	*very high*	32.7	-94.5	-42.0
M1	8	*medium*	35.4	-51.4	-88.0
M2	35	*medium*	47.4	-51.7	-64.7
M3	34	*medium*	43.1	-62.7	-100
M4	14	*medium*	25.8	-18.5	-100
M5	25	*medium*	47.2	-100	-81.0
L1	3	*low*	45.6	-12.6	-48.9
VL1	15	*very low*	61.0	-37.7	-65.7
VL2	19	*very low*	58.9	NA	NA
VL3	5	*very low*	56.9	+28.3*	-96.9
		**Mean:**	**38.3**	**-52.0**	**-75.0**

*This site started with only 1.23% *P. ryukyuensis* cover before the event and ended with 1.57% cover after the event, and likely does not indicate a biologically relevant increase.

Supplementary Table 2 | Environmental parameters on Kiritimati. Pre-bleaching coral cover was assessed using benthic photographs analyzed in CoralNet; no 'before' data was available for *high* disturbance sites. *In situ* salinity, pH, and dissolved oxygen (DO) saturation were measured using a YSI Pro Plus handheld multiparameter meter. Remotely sensed wave energy and maximum and mean net primary productivity was extracted from the open source data product Marine Socio-Environmental Covariates (see Supplementary Methods).

**Disturbance Category**	**Coral Cover (%)**
very high	12.6 ± 13.7
high	NA
medium	38 ± 4.3
low	46.7 ± 0
very low	59.0 ± 3.7

**Disturbance Category**	**Salinity (ppt)**	**pH**	**DO Saturation (%)**
very high	35.6 ± 0.8	7.97 ± 0.08	87 ± 6.6
high	35.4 ± 0.6	7.92 ± 0.04	91 ± 3.9
medium	35.6 ± 0.8	7.92 ± 0.22	92 ± 5.9
low	35.2 ± 0.5	8.00 ± 0.09	89 ± 9.3
very low	35.1 ± 1.4	7.96 ± 0.07	88 ± 6.3

**Disturbance Category**	**NPP Mean (mg C m^{-2} day^{-1})**	**NPP Max (mg C m^{-2} day^{-1})**	**Wave Energy (kW m^{-1})**
very high	908 ± 18	1122 ± 26	25.0 ± 0
high	933 ± 0	1059 ± 0	25.4 ± 0
medium	947 ± 27	1058 ± 31	24.8 ± 0.19
low	880 ± 10	1017 ± 22	25.5 ± 0.19
very low	879 ± 18	1018 ± 16	25.4 ± 0.72

Supplementary Table 3 | Models testing effect of symbiont community on survival status of colonies. S = survival status (alive/dead; confirmed as of March 2016), D = proportion *Durusdinium* and ASV dist = distance matrix of symbiont assemblages (weighted unifrac with ASVs). Symbiont community was quantified in August 2014, January 2015, and May 2015; if a colony was sampled more than once during these three field seasons, only the sample closest to the bleaching event was included to prevent pseudoreplication (i.e., if all a colony was sampled at all three time points, only May 2015 was included in the analysis). Model df = 1 in all cases. Significance is indicated with asterisks as follows: * < 0.05, ** < 0.01, *** < 0.001.

**Model**	**Assumption set (AS)**	**Test Statistic**	**Residual degrees of freedom (df)**	***P*-value**
Platygyra ryukyuensis				
Logistic regression<br>(S ~ D)	AS 1	Z = -3.235	df = 38	*P* = 0.001**
CAP model<br>(ASV dist ~ S)	AS 1	F = 23.85	df = 37	*P* < 0.001***
Favites pentagona				
Logistic regression<br>(S ~ D)	AS 1	Z = -1.197	df = 34	*P* = 0.2310
CAP model<br>(ASV dist ~ S)	AS 1	F = 0.641	df = 34	*P* = 0.417

Supplementary Table 4 | Initial coral symbionts and local disturbance. Coral colonies classified by their dominant (> 50% of reads) Symbiodiniaceae genus (*Cladocopium* (*C.* in table) or *Durusdinium (D.* in table)) and by disturbance level (very high, high, medium, low, very low), prior to the 2015-2016 El Niño (August 2014-May 2015). Almost all corals exposed to *very high* disturbance were dominated by *Durusdinium*, whereas the reverse is true for corals exposed to lower disturbance levels. Additional colonies that were tracked, but had an unknown starting genus, because the initial expedition in which they were tagged and sampled was after the start of the heat stress (e.g., July 2015 or March 2016), are not included in this table, but are as follows: *P. ryukyuensis*, *n* = 22; *F. pentagona*, *n* = 16.

**Disturbance level**	***Very High***		***Medium***		***Low***		***Very Low***		**Overall**	
**Symbiodiniaceae genus**	***C.***	***D.***	***C.***	***D.***	***C.***	***D.***	***C.***	***D.***	***C.***	***D.***
**Coral Species**										
P. ryukyuensis (*n* = 59)	3	16	22	3	4	1	10	0	39 (66%)	20 (34%)
F. pentagona (*n* = 44)	1	15	13	1	4	0	10	0	28 (64%)	16 (36%)
Total (*n* = 103)	4 (11%)	31 (89%)	35 (90%)	4 (10%)	8 (89%)	1 (11%)	20 (100%)	0 (0%)	67 (65%)	36 (35%)

Supplementary Table 5 | Statistical results from constrained ordination analyses of samples before the bleaching event. “Before” the bleaching event included August 2014, January 2015, and May 2015; if a colony was sampled more than once during these three field seasons, only the sample closest to the bleaching event was included to prevent pseudoreplication (i.e., if all a colony was sampled at all three time points, only May 2015 was included in the analysis). capscale results correspond with Fig. 1b, c. Ordistep models initialized with disturbance + region, but the best model only included disturbance for both coral species.

Test	Permutations	Analysis Method	Model (Term)	df	F	*P*
Platygyra ryukyuensis						
PERMANOVA (capscale)	999	ASV	Model: ASV ~ disturbance + leeward/windward	4	19.3	<0.001
			Term: disturbance	3	25.5	<0.001
			Term: leeward/windward	1	0.42	0.516
PERMANOVA (ordistep)	999	ASV	Model: ASV ~ disturbance	3	25.8	<0.001
Favites pentagona						
PERMANOVA (capscale)	999	ASV	Model: ASV ~ disturbance + leeward/windward	4	54.7	<0.001
			Term: disturbance	3	72.9	<0.001
			Term: leeward/windward	1	0.31	0.588
PERMANOVA (ordistep)	999	ASV	Model: ASV ~ disturbance	3	74.2	<0.001

Supplementary Table 6 | Outputs from logistic regression models comparing the effect of environmental parameters on coral symbiont identity. Each model is a quasibinomial logistic regression model with proportion *Durusdinium* as a response variable, including both human disturbance and each additional parameter as explanatory variables. Significant *P*-values are in bold.

Model	Parameter	df error	t value	*P*-value
Platygyra ryukyuensis				
~Disturbance	Intercept	57	-5.427	**<0.0001**
	Disturbance	57	5.195	**<0.0001**
~ Salinity + Disturbance	Intercept	57	-0.390	0.698
	Salinity	57	0.221	0.826
	Disturbance	57	5.144	**<0.0001**
~ pH + Disturbance	Intercept	57	1.391	0.170
	pH	57	-1.504	0.138
	Disturbance	57	5.363	**<0.0001**
~ DO saturation + Disturbance	Intercept	57	0.281	0.7794
	DO saturation	57	-0.076	0.9394
	Disturbance	57	4.103	**0.0001**
~ Wave exposure + Disturbance	Intercept	33	0.709	0.4837
	Wave exposure	33	-0.856	0.3985
	Disturbance	33	2.480	**0.0188**
~ NPP mean + Disturbance	Intercept	57	-1.915	0.0606
	NPP mean	57	1.524	0.1331
	Disturbance	57	5.341	**<0.0001**
~ NPP max + Disturbance	Intercept	57	-2.224	**0.0302**
	NPP max	57	1.833	0.0721
	Disturbance	57	2.457	**0.0171**
Favites pentagona				
~Disturbance	Intercept	48	-3.641	**0.0007**
	Disturbance	48	3.945	**0.0003**

~ Salinity + Disturbance	Intercept	48	0.914	0.366
	Salinity	48	-1.034	0.307
	Disturbance	48	4.693	**< 0.0001**
~ pH + Disturbance	Intercept	48	-0.84	0.405
	pH	48	0.774	0.443
	Disturbance	48	4.33	**< 0.0001**
~ DO saturation + Disturbance	Intercept	48	0.994	0.3253
	DO saturation	48	-1.336	0.1881
	Disturbance	48	4.108	**0.0002**
~ Wave exposure + Disturbance	Intercept	24	0.277	0.7844
	Wave exposure	24	-0.600	0.5548
	Disturbance	24	2.459	**0.0223**
~ NPP mean + Disturbance	Intercept	48	0.786	0.436
	NPP mean	48	-1.176	0.246
	Disturbance	48	4.981	**<0.0001**
~ NPP max + Disturbance	Intercept	48	-1.692	0.0974
	NPP max	48	1.382	0.1735
	Disturbance	48	2.474	**0.0171**

Supplementary Table 7 | Coral symbionts and bleaching. Number of colonies sampled in July 2015 by their bleaching status, classified by dominant Symbiodiniaceae genus (>50% of reads). Sample sizes of colonies that were tracked, but were not sampled in July 2015, and hence are not included in this table, are as follows: *P. ryukyuensis*, *n* = 47; *F. pentagona*, *n* = 27.

**Symbiodiniaceae genus**	***Cladocopium***		***Durusdinium***		***Overall***	
**Bleaching status**	**Bleached**	**Healthy**	**Bleached**	**Healthy**	**Bleached**	**Healthy**
**Coral Species**						
P. ryukyuensis (*n* = 34)	19 (79%)	5 (21%)	4 (40%)	6 (60%)	23 (68%)	11 (32%)
F. pentagona (*n* = 33)	17 (77%)	5 (23%)	3 (27%)	8 (73%)	20 (61%)	13 (39%)
Total across species (*n* = 67)	36 (78%)	10 (22%)	7 (33%)	14 (67%)	43 (64%)	24 (36%)

Supplementary Table 8 | Visual assessment of bleaching and recovery in July 2015 and March 2016. Shown are the number of surviving colonies that were photographed at both time points and found to have recovered, had their condition worsen, or were assigned to the same bleaching status at both timepoints. Colonies sampled in only one of these time points are not shown, including when the colony was found dead. Several of the *Platygyra ryukyuensis* colonies classified as "same" in fact showed increased symbiont-to-host ratios, despite their visually assessed bleaching status not improving

Species	Disturbance	Recovered	Worsened	Same
Platygyra ryukyuensis	very low	4	1	5
(n = 20)	low	0	0	0
	medium	8	0	1
	very high	0	0	1
Favites pentagona (n = 11)	very low	2	0	1
	low	0	0	1
	medium	2	0	0
	very high	1*	2	2

*This colony was only marginally bleached in July 2015 and its recovery is a consequence of partial mortality of the bleached part of the colony, amounting to less than 10% partial mortality overall but a "recovered" status

Supplementary Table 9 | Human disturbance calculations and leeward/windward classifications for Kiritimati atoll. Number of people residing within 2 km of each site (HumanPop), calculated based on Kiribati Population Census report[6]. FishPressure is a kernel density function of fishing intensity with ten discrete levels (from Watson *et al.*[7]). LocalDisturb is the sum of the previous two columns. sqrt(LocalDisturb) is the square root of LocalDisturb, and is used in all models with disturbance as a continuous variable. Based on these calculations, sites are categorized into five distinct human disturbance categories. Site Name is included for cross-comparisons with other related Kiritimati publications.

Site Name	HumanPop	FishPressure	LocalDisturb	sqrt(LocalDisturb)	Disturbance Category	Leeward/ Windward
VH1	4042	3234	7276	85	very high	leeward
VH2	1223	3638	4861	70	very high	leeward
VH3	3065	2021	5086	71	very high	leeward
H1	0	2425	2425	49	high	leeward
M1	0	1213	1213	35	medium	leeward
M2	0	1213	1213	35	medium	leeward
M3	0	1213	1213	35	medium	leeward
M4	351	809	1160	34	medium	leeward
M5	0	1617	1617	40	medium	windward
L1	0	809	809	28	low	windward
L2	0	809	809	28	low	windward
VL1	0	0	0	0	very low	windward
VL2	0	0	0	0	very low	windward
VL3	0	0	0	0	very low	leeward
VL4	0	0	0	0	very low	leeward

Supplementary Table 10 | A comparison of *in situ* versus NOAA maximum degree heating weeks (DHW) for each region. See Claar *et al.*[2] for more details of methodology.

Region	NOAA max DHWs	*In situ* max DHWs with local MMM offset	*In situ* max DHWs with NOAA MMM
Vaskess Bay	25.3	31.7	27.2
South Lagoon	24.4	33.5	26.2
Mid Lagoon	24.3	33.0	26.0
North Lagoon	24.5	34.0	26.0
North Shore	24.5	NA*	NA*
Bay of Wrecks	25.3	26.2	27.0

******in situ* loggers were not present for the entire duration of the event in this region, thus we could not calculate *in situ* maximum thermal stress.

Supplementary Table 11 | Tagged colony sample sizes by site. Shown are the numbers of colonies that were tagged and followed at each site. Also shown are the expeditions that each site was sampled (*i* = August 2014, *ii* = January 2015, *iii* = May 2015, *iv* = July 2015, *vi* = March 2016, *vii* = November 2016, *viii* = July 2017).

**Site Name**	**Site ID**	**Disturbance Category**	***Platygyra ryukyuensis***	***Favites pentagona***	**Expeditions sampled**
VH1	27	very high	*n* = 9	*n* = 10	i, ii ,iii, iv, vi, vi, viii
VH2	32	very high	*n* = 7	*n* = 8	i, ii ,iii, iv, vi, vi, viii
VH3	30	very high	*n* = 3	*n* = 3	i, ii ,iii, iv, vi, vi, viii
H1	40	high	*n* = 3	*n* = 4	vi, vii, viii
M1	8	medium	*n* = 8	*n* = 4	i, ii ,iii, iv, vi, vi, viii
M2	35	medium	*n* = 5	*n* = 2	i, ii ,iii, iv, vi, vi, viii
M3	34	medium	*n* = 9	*n* = 6	i, iii, iv, vi, vi, viii
M4	14	medium	*n* = 5	*n* = 2	i, vi
M5	25	medium	*n* = 4	*n* = 3	i, iv, viii
L1	3	low	*n* = 6	*n* = 1	i, iv, viii
L2	38	low	*n* = 1	*n* = 3	i, iv, vi, vii
VL1	15	very low	*n* = 3	*n* = 9	ii ,iii, iv, vi, vi, viii
VL2	19	very low	*n* = 0	n = 1	iv, vii
VL3	5	very low	*n* = 7	*n* = 2	iii, iv, vi, vii, viii
VL4	37	very low	*n* = 11	*n* = 2	vi, vii, viii
		**Total:**	***n* = 81**	***n* = 60**	

Supplementary Table 12 | Sensitivity of analyses to assumptions about survival status. We conducted survival analyses (Logistic regressions and constrained ordination (CAP) models) on three different sets of assumptions for evaluating survival. The results of assumption set 1 are present in the main text: skeleton found and colony dead = “dead”, colonies missing completely (“gone”) were considered “dead” if seen during the previous expedition, otherwise they were excluded. Assumption set 2: exclude all colonies recorded as “gone” from analysis; Assumption set 3: all “gone” colonies considered “dead”. S = survival status (alive/dead), D = proportion *Durusdinium* and ASV dist = distance matrix of symbiont assemblages (weighted unifrac with ASVs). Model df = 1 in all cases. Significance is indicated with asterisks as follows: * < 0.05, ** < 0.01, *** < 0.001.

Model	Assumption set (AS)	Test Statistic	Residual degrees of freedom (df)	P-value
Platygyra ryukyuensis				
Logistic regression	AS 1	Z = -3.235	df = 38	$P = 0.001$**
(S ~ D)	AS 2	Z = -3.111	df = 29	$P = 0.002$**
	AS 3	Z = -2.709	df = 41	$P = 0.006$**
CAP model	AS 1	F = 23.85	df = 37	$P < 0.001$***
(ASV dist ~ S)	AS 2	F = 49.132	df = 29	$P < 0.001$***
	AS 3	F = 13.215	df = 42	$P < 0.001$***
Favites pentagona				
Logistic regression	AS 1	Z = -1.197	df = 34	$P = 0.231$
(S ~ D)	AS 2	Z = -0.517	df = 26	$P = 0.605$
	AS 3	Z = -1.069	df = 35	$P = 0.285$
CAP model	AS 1	F = 0.641	df = 34	$P = 0.417$
(ASV dist ~ S)	AS 2	F = 0.084	df = 26	$P = 0.953$
	AS 3	F = 0.460	df = 35	$P = 0.525$

Supplementary Table 13 | Partial mortality in surviving colonies of both species by dominant symbiont. Shown are the numbers of surviving colonies that were resampled in 2016 (either March or November) and the estimated percent lost through partial mortality through the heatwave – determined by comparing to photos from July 2015 or earlier.

**Symbiodiniaceae genus**	***Cladocopium***			***Durusdinium***		
**Partial mortality**	**< 10%**	**11-49%**	**> 50%**	**< 10%**	**11-49%**	**> 50%**
**Coral Species**						
P. ryukyuensis (*n* = 21)	10	7	3	0	0	1
F. pentagona (*n* = 15)	1	4	4	6	0	0

Supplementary Figures

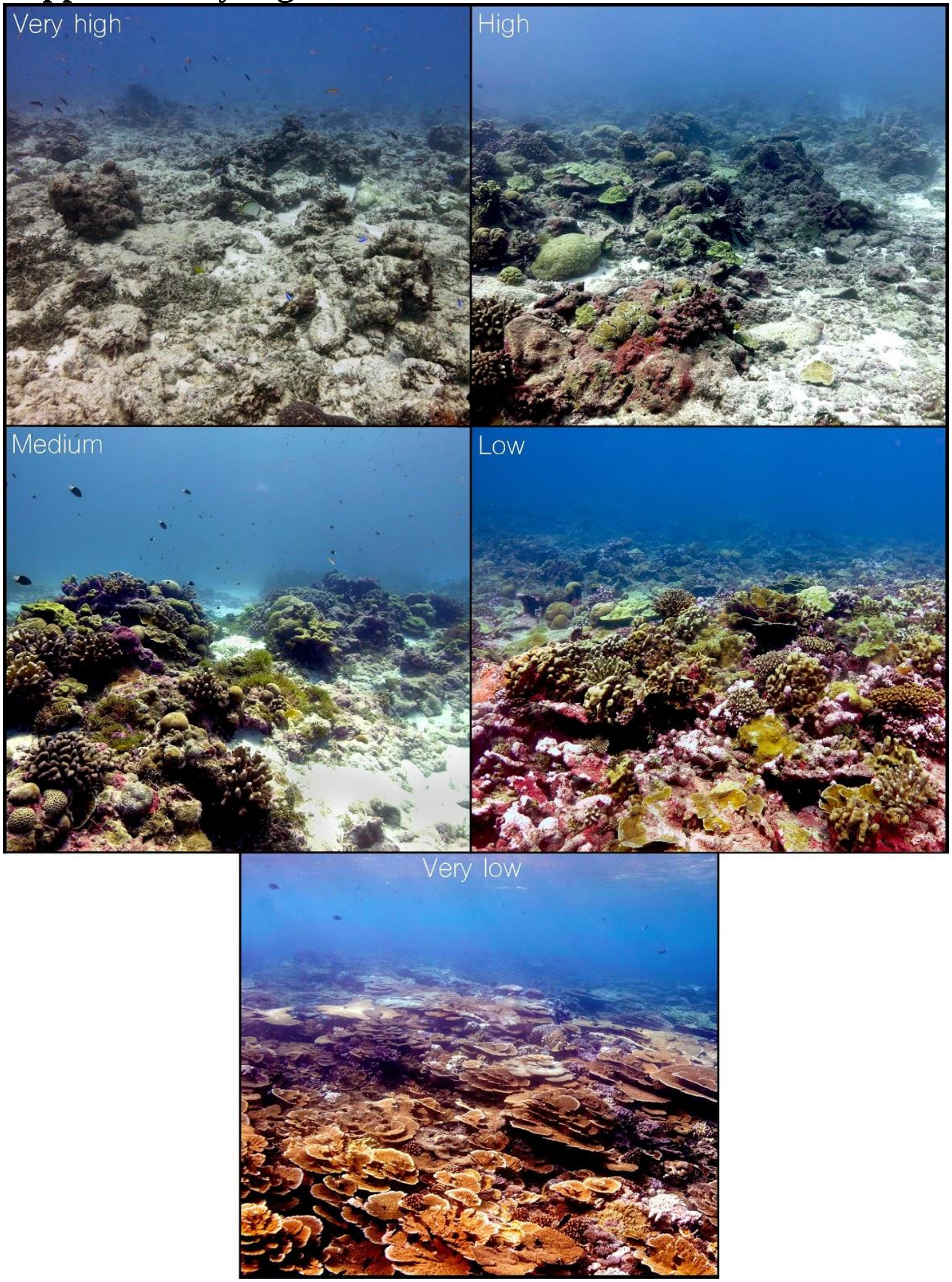

Supplementary Figure 1 | Human disturbance gradient on Kiritimati. Photographs from before the 2015-2016 El Niño, at sites on Kiritimati representing each of the atoll's five different levels of local human disturbance.

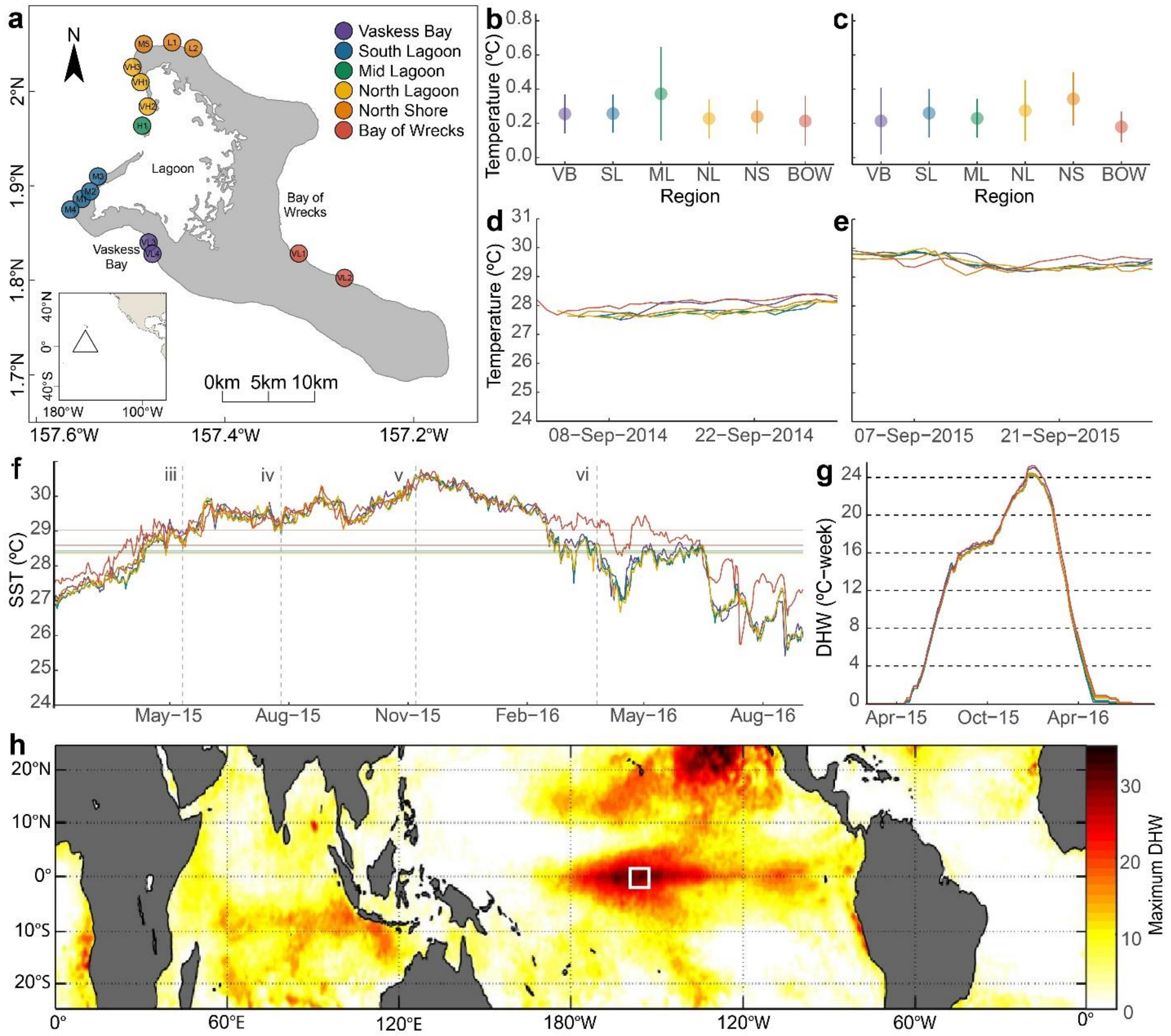

Supplementary Figure 2 | Temperature across Kiritimati atoll at different spatial and temporal resolutions. Panel **a** shows the six regions for which local temperatures were separately estimated using *in situ* temperature loggers in **b-g**. Panels **b**, **c** show daily variation (range) in temperature per region both before (2014-09-09 to 2015-06-09), **b**, and during (2015-06-09 to 2016-04-26), **c.**, the heatwave. Error bars represent standard deviation. Sample sizes of individual hourly temperature measurements are as follows (from left to right): **b**, n = 274, n = 272, n = 274, n = 273, n = 274, n = 274, **c.**, n = 323, n = 323, n = 323, n = 321, n = 149, n = 323. Panels **d, e** show an example of 3 week periods before, **d**, or during, **e**, the heatwave. **f**, Regional temperatures through the heatwave with the six local bleaching threshold values color coded by region and shown as straight horizontal lines. Some regions are so similar that they overlap completely. Although there were differences in bleaching threshold amongst regions of the reef, all regions had temperatures above or fluctuating around their bleaching thresholds during our expedition in March/April 2016 (time point vi). **g**, Regional thermal stress (degree heating weeks, DHW) given by the NOAA 5-km product. NOAA's degree heating week product is so similar between regions that it overlaps completely in most cases (see [1] with comparisons to between *in situ* and satellite temperature data). Panel **h** shows the distribution of thermal stress

through the 2015-2016 El Nino across the Pacific. The location of Kiritimati is indicated with a small square.

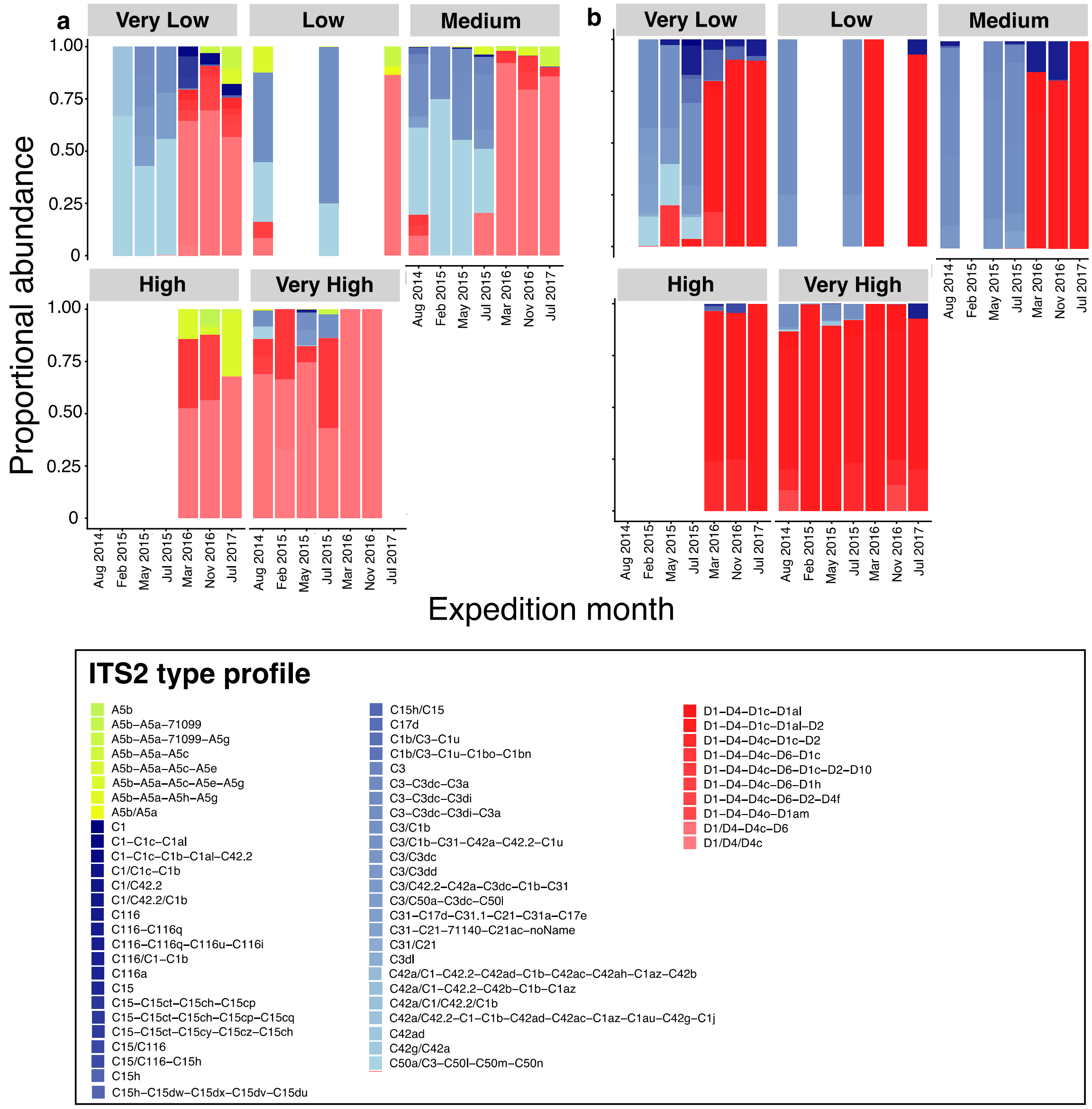

Supplementary Figure 3 | ITS2 type profiles for corals sampled during each expedition. Stacked bar plots show the relative number of sequence reads assigned to each ITS2 type profile (as determined by SymPortal) averaged across individual colonies within each disturbance category. Panel **a** shows data for *Platygyra ryukyuensis* and panel **b** shows data for *Favites pentagona*. Also shown is a legend (bottom panel) of different ITS2 type profiles. Profiles starting in A, C and D represent taxa from the genera *Symbiodinium*, *Cladocopium* and *Durusdinium*, respectively. *Durusdinium* profiles likely represent the taxa *D. glynnii* and *D. trenchii*.

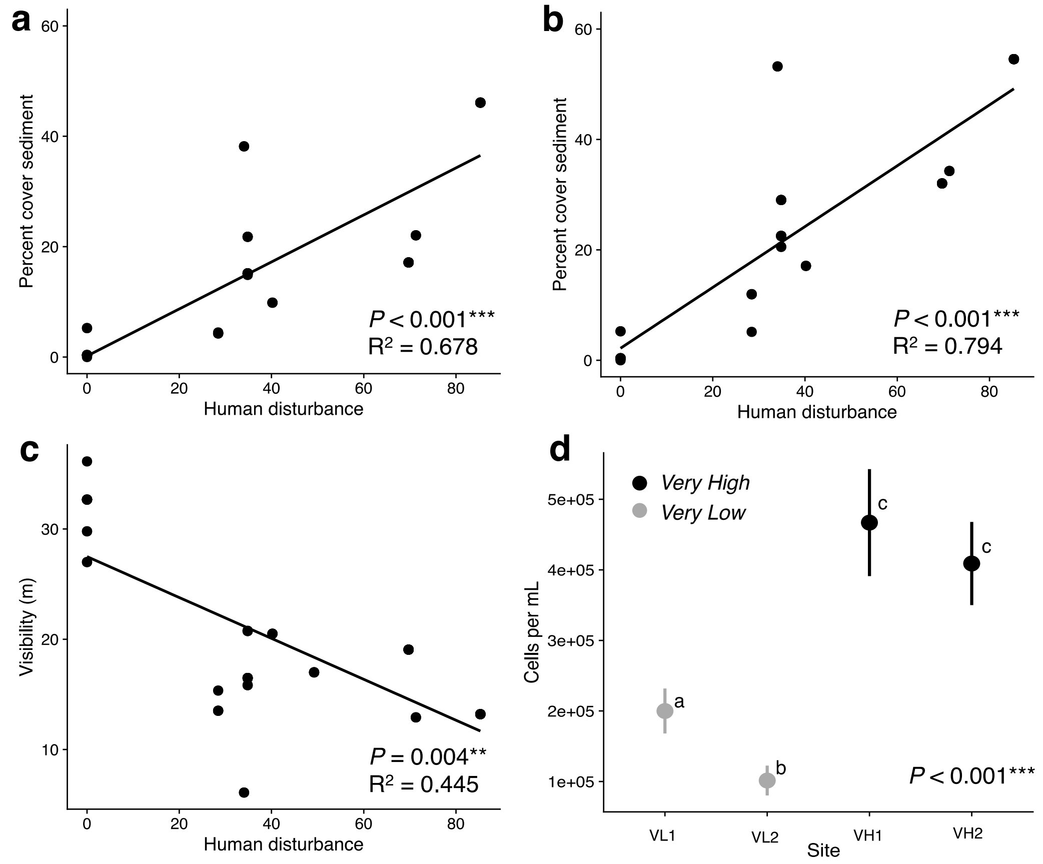

Supplementary Figure 4. Indicators of human disturbance across Kiritimati atoll. a, b, Relationship between benthic sediment cover, both without (**a**) and with (**b**) sand included and human disturbance index (sqrt(LocalDisturb); Supplementary Table 7). **c,** Relationship between water column visibility (a proxy of turbidity) and human disturbance index. **d,** A comparison of microbial counts at two very high disturbance and two very low disturbance sites. Letters indicate significant differences between means as determined by a Tukey post-hoc test. Data in panel d are from McDevitt-Irwin *et al.*[3], error is shown as standard deviation, $n = 4$ samples per site.

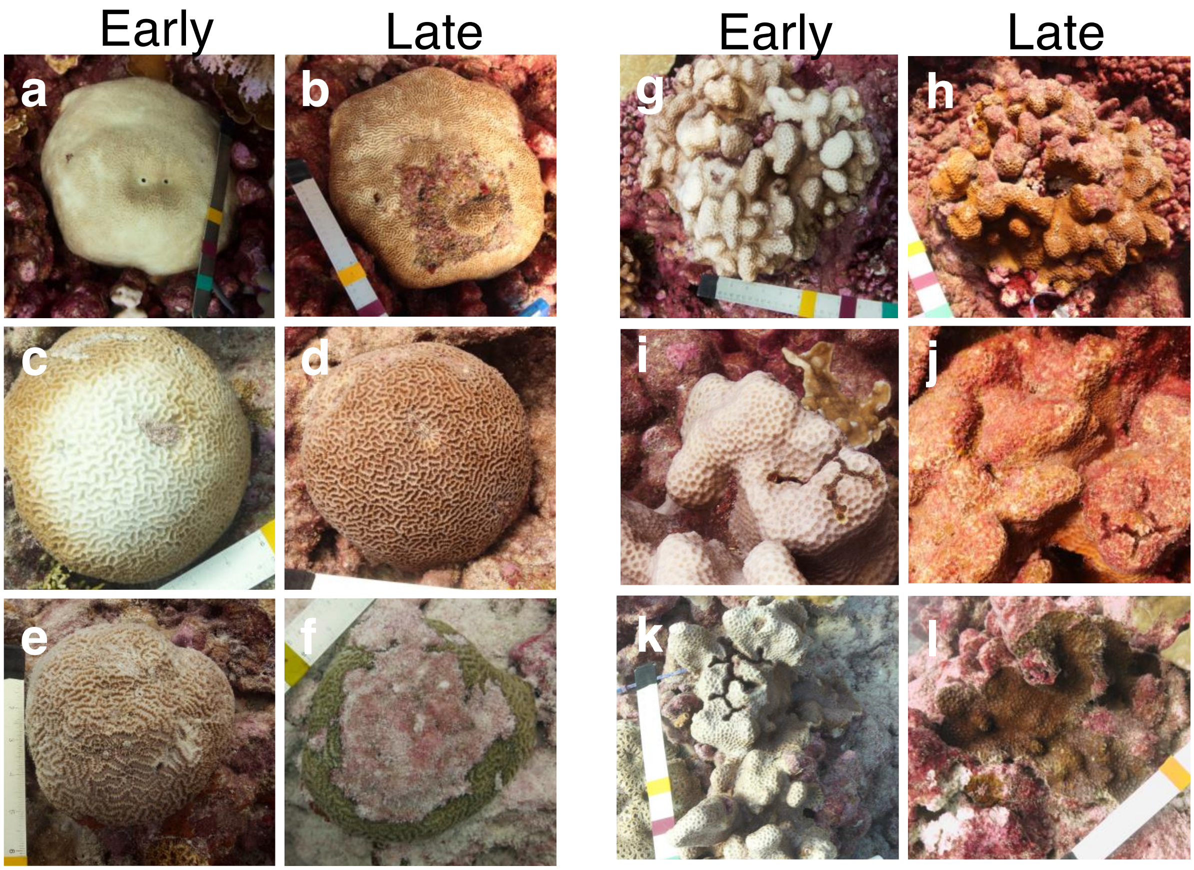

Supplementary Figure 5 | Examples of colonies that were bleached early (July 2015) in the heatwave but were recovered at the time of our sampling late (March/April 2016) in the event. a-f: *Platygyra ryukyuensis*; **g-l:** *Favites pentagona.* **a, b** and **g-j** show colonies from a *very low* disturbance site in the Bay of Wrecks, while the remaining panels are from *medium* disturbance sites. Pictures are paired such that images labelled as "early" and "late" in a given row are the same colony. Colony shown in **e-f** is presented as an example of as *P. ryukyuensis* colonies that experienced partial mortality (approximately 50% of surviving *P. ryukyuensis* colonies experienced greater than 10% partial mortality).

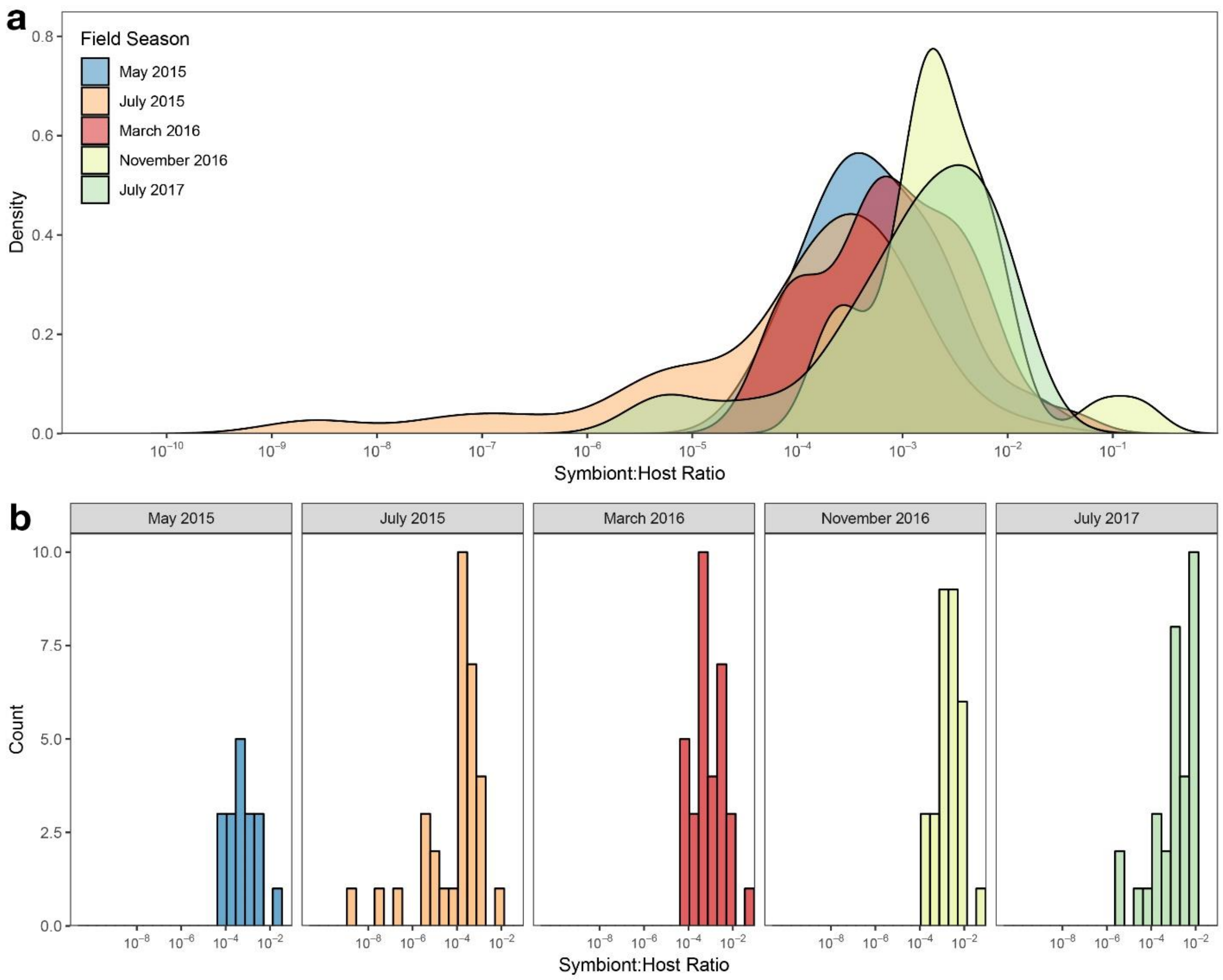

Supplementary Figure 6 | Total symbiont-to-host cell ratio (S:H) for all *Platygyra ryukyuensis* samples (i.e., from all colonies and field seasons) through the 2015-2016 El Niño event colored by field season. These plots show how the density of Symbiodiniaceae within corals varied over time. **a** Density plot by field season, showing changes in the distribution of S:H throughout the El Niño event. There is a longer tail to the left during July 2015, with lower S:H during peak bleaching demonstrating bleached coral colonies. **b** Histograms of S:H cell ratio by field season, showing the same trend. Sample sizes are as follows: May 2015, n = 18; July 2015, n = 32; March 2016, n = 33; November 2016, n = 32; July 2017, n = 31.

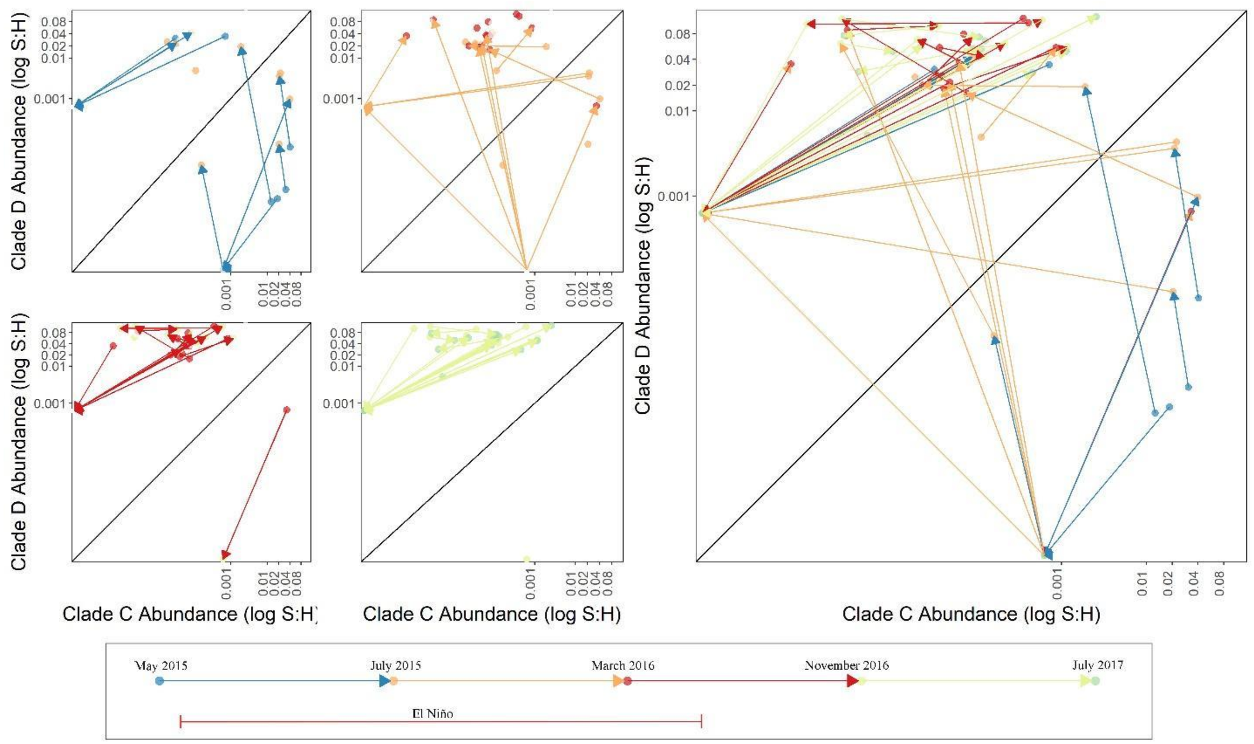

Supplementary Figure 7 | Trajectories of the Symbiodiniaceae communities of all individual *Platygyra* coral colonies through the 2015-2016 El Niño event. Each point represents the Symbiodiniaceae community of a single coral colony, and each arrow shows the shift from the initial point to the new Symbiodiniaceae community at the next timestep. Left panels: Symbiodiniaceae community changes between single time points (blue arrows, May to July 2015; orange arrows, July 2015 to March 2016; red arrows, March to November 2016; green arrows, November 2016 to July 2017) showing the transition from *Cladocopium* dominated communities to *Durusdinium* dominated communities during the El Niño (first two time points), and the stability of *Durusdinium* dominated communities after the El Niño (last two time points). Right panel: Symbiodiniaceae community change for all time periods combined. Sample sizes are as follows: May 2015, n = 18; July 2015, n = 32; March 2016, n = 33; November 2016, n = 32; July 2017, n = 31.

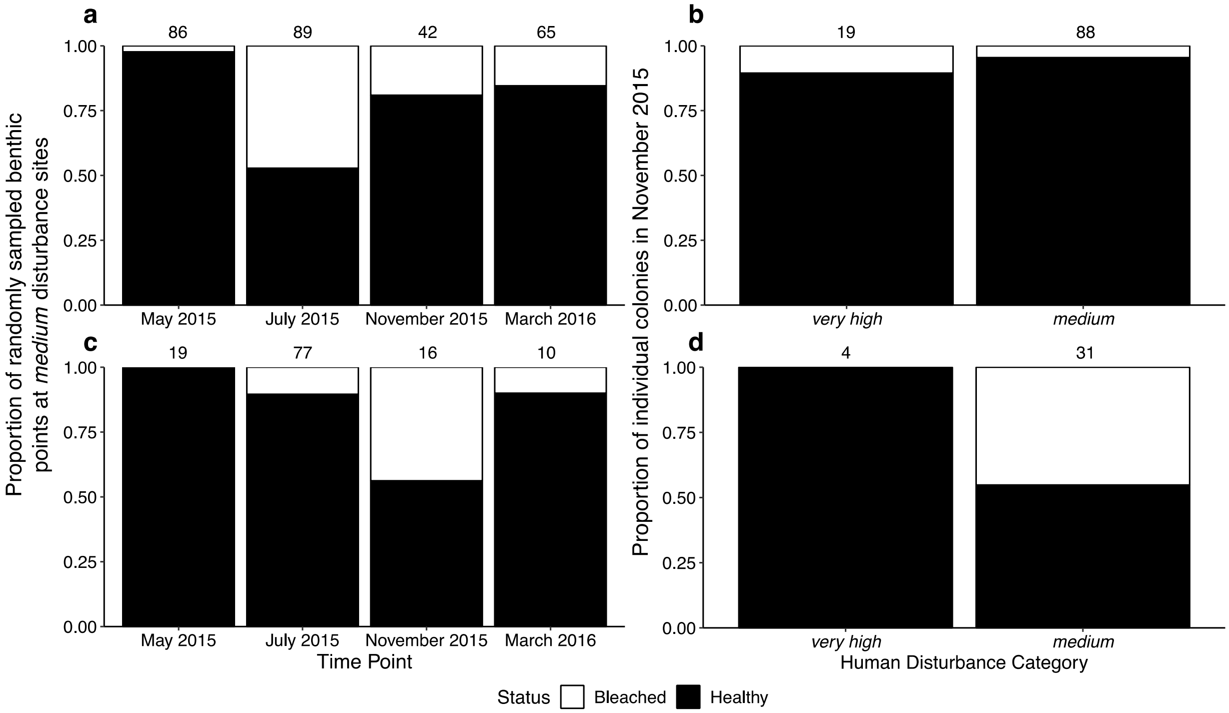

Supplementary Figure 8 | Timing of bleaching in *Platygyra ryukyuensis* and *Favites pentagona* through the heatwave, as determined by photo analyses. a, **c** show the proportion of random points (from CoralNet analyses of benthic photoquadrats) that were bleached versus healthy at each of four sampling time points, for the two *medium* disturbance sites that were sampled in November 2015. Note that although the November 2015 expedition also collected photoquadrat data at two *very high* disturbance sites, they did not contain enough points of either species (< 5) to be included. **b, d** show the proportion of individual coral colonies that were bleached versus healthy in November 2015 as determined from a haphazard sampling of site photos (see Supplementary Methods) for the four sites that were visited. **a, b:** *Platygyra ryukyuensis*; **c, d:** *Favites pentagona*. Sample sizes (number of points **a, c** or colonies **b**, **d**) are shown above each stacked bar.

Supplementary References

1. Yeager, L. A., Marchand, P., Gill, D. A., Baum, J. K. & McPherson, J. M. Marine Socio-Environmental Covariates: queryable global layers of environmental and anthropogenic variables for marine ecosystem studies. *Ecology* **98,** 1976 (2017).
2. Claar, D. C., Cobb, K. M. & Baum, J. K. In situ and remotely sensed temperature comparisons on a Central Pacific atoll. *Coral Reefs* **38**, 1343–1349 (2019).
3. McDevitt-Irwin, J. M., Garren, M., McMinds, R., Vega Thurber, R. & Baum, J. K. Variable interaction outcomes of local disturbance and El Niño-induced heat stress on coral microbiome alpha and beta diversity. *Coral Reefs* **38**, 331–345 (2019).
4. NOAA Coral Reef Watch Version 3.1, Daily Global 5-km Satellite Coral Bleaching Degree Heating Week Product, August 11 to November 21, 1998, July 21 to November 11, 2016. College Park, Maryland, USA: NOAA Coral Reef Watch. *Data set https://coralreefwatch. noaa. gov/satellite/bleaching5km/index. php (accessed 16 November 2018)* (2018).
5. Claar, D. C., Szostek, L., McDevitt-Irwin, J. M., Schanze, J. J. & Baum, J. K. Global patterns and impacts of El Niño events on coral reefs: A meta-analysis. *PLoS One* **13**, (2018).
6. Morate, O. *2015 Population and Housing Census. Volume 1: Management Report and Basic Tables*. (2016).
7. Watson, M. S., Claar, D. C. & Baum, J. K. Subsistence in isolation: Fishing dependence and perceptions of change on Kiritimati, the world’s largest atoll. *Ocean Coast. Manag.* **123**, 1–8 (2016).
