# Supplementary material for: Dynamic symbioses reveal pathways to coral survival through prolonged heatwaves
Source: Nat Commun. 2020 Dec 8;11:6097. doi: 10.1038/s41467-020-19169-y (PMC7723047; doi:10.1038/s41467-020-19169-y)
Supplement: Supplementary file 2 — Reporting Summary [file 41467_2020_19169_MOESM2_ESM.pdf]

nature research

Corresponding author(s): Julia K. Baum

Last updated by author(s): Sep 2, 2020

Reporting Summary

Nature Research wishes to improve the reproducibility of the work that we publish. This form provides structure for consistency and transparency in reporting. For further information on Nature Research policies, see our Editorial Policies and the Editorial Policy Checklist.

Statistics

For all statistical analyses, confirm that the following items are present in the figure legend, table legend, main text, or Methods section.

n/a	Confirmed	
[ ]	[x]	The exact sample size (*n*) for each experimental group/condition, given as a discrete number and unit of measurement
[ ]	[x]	A statement on whether measurements were taken from distinct samples or whether the same sample was measured repeatedly
[ ]	[x]	The statistical test(s) used AND whether they are one- or two-sided<br>*Only common tests should be described solely by name; describe more complex techniques in the Methods section.*
[ ]	[x]	A description of all covariates tested
[ ]	[x]	A description of any assumptions or corrections, such as tests of normality and adjustment for multiple comparisons
[ ]	[x]	A full description of the statistical parameters including central tendency (e.g. means) or other basic estimates (e.g. regression coefficient) AND variation (e.g. standard deviation) or associated estimates of uncertainty (e.g. confidence intervals)
[ ]	[x]	For null hypothesis testing, the test statistic (e.g. *F*, *t*, *r*) with confidence intervals, effect sizes, degrees of freedom and *P* value noted<br>*Give P values as exact values whenever suitable.*
[x]	[ ]	For Bayesian analysis, information on the choice of priors and Markov chain Monte Carlo settings
[ ]	[x]	For hierarchical and complex designs, identification of the appropriate level for tests and full reporting of outcomes
[x]	[ ]	Estimates of effect sizes (e.g. Cohen's *d*, Pearson's *r*), indicating how they were calculated

Our web collection on statistics for biologists contains articles on many of the points above.

Software and code

Policy information about availability of computer code

Data collection	Data collection occurred in the field, and did not have any associated code.
Data analysis	We used the SymPortal bioinformatics framework (Hume et al. 2019; version 0.3.18) for sequence filtering and analysis, as well as ASV analysis of microbial data. All statistical analyses were conducted in R version 3.4.3. The following software was used: CoralNet (beta version), dada2 v1.12.1 (R); phyloseq v1.28.0 (R); phangorn 2.5.5 (R); DECIPHER v2.12.0 (R); vegan v2.5-5 (R); arm v1.10-1 (R); lme4 v1.1-21 (R); emmeans v1.3.5 (R)

For manuscripts utilizing custom algorithms or software that are central to the research but not yet described in published literature, software must be made available to editors and reviewers. We strongly encourage code deposition in a community repository (e.g. GitHub). See the Nature Research guidelines for submitting code & software for further information.

Data

Policy information about availability of data

All manuscripts must include a data availability statement. This statement should provide the following information, where applicable:

- Accession codes, unique identifiers, or web links for publicly available datasets
- A list of figures that have associated raw data
- A description of any restrictions on data availability

All data that support the results of this study are available on GitHub (https://github.com/baumlab/Claar_etal_2020_NatCom) with the following identifier: https://doi.org/10.5281/zenodo.4014057. We used previously collected data for the PaxC intron of P. ryukyuensis from NCBI Nucleotide database (accession numbers KX026897 - KX026910) to develop qPCR probes. .Next-generation sequencing data are available in the NCBI Sequence Read Archive Accession Number PRJNA543540.

April 2020

Field-specific reporting

Please select the one below that is the best fit for your research. If you are not sure, read the appropriate sections before making your selection.

☐ Life sciences ☐ Behavioural & social sciences ☒ Ecological, evolutionary & environmental sciences

For a reference copy of the document with all sections, see nature.com/documents/nr-reporting-summary-flat.pdf

Ecological, evolutionary & environmental sciences study design

All studies must disclose on these points even when the disclosure is negative.

Study description	Corals were sampled at 15 sites across five levels of local human disturbance. Corals were tagged for re-sampling, and samples were collected before, during, and at the end of the major 2015/2016 El Niño event that caused ten months of sustained temperature stress on Kiritimati atoll. Benthic photographs were used to quantify coral cover loss due to the bleaching event. For the focal coral species, qPCR was used to quantify symbiont abundances, and for both species Illumina MiSeq was used to determine symbiont sequence identity. The study design was nested, in that corals were collected from individual sites that were nested within the human disturbance gradient, and samples were taken at multiple time points.
Research sample	The focal coral species were Platygyra ryukyuensis and Favites pentagona. We tagged and sampled coral colonies of two species (n = 141 total; Fig. 4) along a 60 m transect, laid along the 10-12 m isobath, at each of 15 different fore reef sites around Kiritimati (Fig. 1), during expeditions before (August 2014, January/February 2015, April/May 2015), during (July 2015, March 2016), and after (November 2016, July 2017) the El Niño heatwave. Two sites (H1, VL4) were sampled for the first time in March 2016 and one site was sampled for the first time in July 2015 (VL2). These sites were therefore not included in analyses presented in Fig. 1. Sample sizes by site are presented in Supplementary Table 1012. Each coral colony was photographed at its initial tagging and at each revisit to record colony measurements and bleaching (see Supplementary Methods). Not all sites could be visited during all field seasons, and some site surveys were only partially completed during some field seasons due to inclement weather conditions.
Sampling strategy	We include data from 141 coral colonies. This sample size is above and beyond what most coral-Symbiodiniaceae studies. Sample size was maximized with respect to weather conditions, available bottom time (i.e., dive safety), and number of days on island.
Data collection	Data were collected by the Baum Lab Kiritimati Island field team. Samples were collected by individuals trained in coral sampling.
Timing and spatial scale	Coral samples were collected in August 2014, January 2015, May 2015, July 2015, March 2016, coinciding with before (August 2014 - May 2015), two months into (July 2015) and ten months into (March 2016) the 2015/2016 El Niño event. Corals were sampled at sites around Kiritimati atoll (see map), based on a long-term monitoring program that began in 2007 and that is currently ongoing at the time of publication.
Data exclusions	During MiSeq sequence QA, samples with fewer than 1,000 sequences were removed before ASV analyses and those with fewer than 250 reads were excluded before ITS2 profile analyses. These cut-offs have been used previously, and are important to exclude samples with spuriously low sequence reads (which can increase the probability of sequencing error).
Reproducibility	The analyses from this paper are reproducible with the code provided at https://github.com/baumlab/Claar_etal_2020_NatCom.
Randomization	Corals were tagged and sampled from pre-established long-term monitoring sites. We have collected data on and accounted for a suite of environmental covariates associated with sites around the atoll.
Blinding	Sequence analyses were conducted via a standard pipeline that did not consider sample origin or characteristics.

Did the study involve field work? ☒ Yes ☐ No

Field work, collection and transport

Field conditions	Data were collected via SCUBA diving, primarily from a small boat but also via shore diving. Water temperature is explained in depth in the manuscript (Figure 2) and the supplementary (Supplementary Figure 2). Field work took place under conditions that were sometimes fair, but often arduous, including rainfall, wind, and swell.
Location	Samples were collected on Kiritimati atoll (Republic of Kiribati) in the central equatorial Pacific Ocean (01°52′N 157°24′W), at 10-12 m water depth.
Access & import/export	All collections were conducted under a Republic of Kiribati, Environment and Conservation Division, Scientific Research Permit numbers 007/14 and 001/16.
Disturbance	Coral samples that were taken were very small (<5 mm squared), and were the minimum necessary for our study. Responsible diving practices were employed throughout the study to avoid any damage to the reef.

Reporting for specific materials, systems and methods

We require information from authors about some types of materials, experimental systems and methods used in many studies. Here, indicate whether each material, system or method listed is relevant to your study. If you are not sure if a list item applies to your research, read the appropriate section before selecting a response.

Materials & experimental systems

n/a	Involved in the study
x	Antibodies
x	Eukaryotic cell lines
x	Palaeontology and archaeology
	x Animals and other organisms
x	Human research participants
x	Clinical data
x	Dual use research of concern

Methods

n/a	Involved in the study
x	ChIP-seq
x	Flow cytometry
x	MRI-based neuroimaging

Animals and other organisms

Policy information about studies involving animals; ARRIVE guidelines recommended for reporting animal research

Laboratory animals	N/A
Wild animals	Corals were micro-sampled (< 5 mm squared tissue removed from each sampled coral colony). All sampling was conducted under a Government of Kiribati permit.
Field-collected samples	Coral samples were collected in the field. They were stored directly on ice, until processing in the evening and long-term storage.
Ethics oversight	No ethics oversight was required, as this study only involved invertebrates.

Note that full information on the approval of the study protocol must also be provided in the manuscript.
